# The yeast Cyc8–Tup1 complex cooperates with Hda1p and Rpd3p histone deacetylases to robustly repress transcription of the subtelomeric *FLO1* gene

**DOI:** 10.1016/j.bbagrm.2014.07.022

**Published:** 2014-11

**Authors:** Alastair B. Fleming, Suzanne Beggs, Michael Church, Yoshihiro Tsukihashi, Sari Pennings

**Affiliations:** a School of Genetics and Microbiology, Trinity College Dublin, College Green, Dublin 2, Ireland; b School of Biomedical Sciences, University of Edinburgh, Edinburgh, EH8 9XD, UK; c Queen’s Medical Research Institute, University of Edinburgh, Edinburgh, EH16 4TJ, UK

**Keywords:** Cyc8 (Ssn6)-Tup1, Gene repression, Chromatin, Histone acetylation, Swi–Snf, *Saccharomyces cerevisiae*

## Abstract

We demonstrate that the yeast flocculation gene, *FLO1*, is representative of a distinct subset of subtelomeric genes that are robustly repressed by the Cyc8–Tup1 complex. We have examined Cyc8–Tup1 localisation, histone acetylation and long-range chromatin remodelling within the extensive *FLO1* upstream region. We show that Cyc8–Tup1 is localised in a DNase I hypersensitive site within an ordered array of strongly positioned nucleosomes around − 700 base pairs upstream of the transcription start site. In *cyc8* deletion mutant strains, Tup1p localisation is absent, with concomitant histone hyperacetylation of adjacent regions at the *FLO1* promoter. This is accompanied by extensive histone depletion across the upstream region and gene activation. The yeast histone deacetylases, Hda1p and Rpd3p, occupy the repressed *FLO1* promoter region in a Cyc8–Tup1 dependent manner and coordinate histone deacetylation, nucleosome stabilisation and gene repression. Moreover, we show that the ATP-dependent chromatin remodelling complex Swi–Snf occupies the site vacated by Cyc8–Tup1 in a *cyc8* mutant. These data suggest that distinctly bound Cyc8–Tup1 cooperates with Hda1p and Rpd3p to establish or maintain an extensive array of strongly positioned, deacetylated nucleosomes over the *FLO1* promoter and upstream region which inhibit histone acetylation, block Swi–Snf binding and prevent transcription.

## Highlights

• Cyc8–Tup1 repression activity is enriched at chromosome subtelomeric regions.
• The subtelomeric *FLO1* gene is subject to chromatin-mediated repression by Cyc8–Tup1.
• Cyc8–Tup1 promotes long-range nucleosome positioning and histone deacetylation.
• Hda1p and Rpd3p cooperate with Cyc8–Tup1 to facilitate this repressive chromatin.
• Swi–Snf directs extensive nucleosome remodelling when Cyc8–Tup1 is absent.

## 1 Introduction

Co-repressors and co-activators play a key role in eukaryotic gene regulation. Through association with DNA-bound transcription factors in the nucleus, these often large complexes determine the repressed or active state of gene promoters by altering the chromatin structure or the recruitment and competence of the RNA polymerase II holoenzyme. One of the earliest co-repressors of gene regulation identified in the yeast, *Saccharomyces cerevisiae*, was the Cyc8–Tup1 complex; a 1.4 megadalton complex composed of the *TUP1* and *CYC8* (also known as *SSN6*) gene products in a 4:1 ratio [1], [2]. The complex is formed by the interaction of the N-terminal residues of Tup1p with tetratricopeptide repeat (TPR) motifs of Cyc8p [3], [4], [5]. Tup1p is related to the Groucho family of co-repressor proteins in higher eukaryotes by way of its C-terminal WD-repeat domain [6].

Cyc8–Tup1 is involved in many pathways in yeast such as glucose, starch and oxygen utilisation, osmotic stress, DNA repair, mating, sporulation, meiosis and flocculation [7], [8]. Recruitment of the Cyc8–Tup1 complex, which has no DNA binding activity, is directed by promoter-specific DNA binding factors such as α2-Mcm1p, Crt1p, Mig1p, Rox1p, and Sfl1p [7], [8], [9]. Intriguingly, Cyc8–Tup1 has been shown to remain at some target gene promoters during activation of transcription [10], [11], [12]. Moreover, there is evidence that the persistence of Cyc8–Tup1 at these genes, and at other genes, is required for gene activation [13], [14], [15].

However, the mechanism of Cyc8–Tup1 repression is still unclear. Cyc8–Tup1 can interact directly with several components of the transcriptional machinery such as the kinase-cyclin pair Srb10/11p, the essential holoenzyme component Srb7p, and the Mediator subunit Med3p [6]. This is consistent with genetic interactions found between Cyc8–Tup1 and RNA polymerase II holoenzyme components [16], [17], [18], [19]. Cyc8–Tup1 has also been proposed to directly block transcription activators [20], [21], [22], [23]. Indeed, recent work suggests the primary role for Cyc8–Tup1 involves obstructing the activation domains of the DNA binding proteins responsible for its targeted recruitment [23].

The Cyc8–Tup1 complex also interacts with chromatin and can influence nucleosome positioning, histone acetylation and deposition of the histone variant, Htz1p [7], [24], [25], [26]. Tup1p binds the deacetylated, but not acetylated, histone tails of H3 and H4 *in vitro*, and the domain that is required for this interaction overlaps with the transcription repression domain [27]. Furthermore, repression of transcription at Cyc8–Tup1 regulated genes is correlated with a reduced acetylation of histone H3 and H4 at promoters *in vivo* [28], [29], [30]. Previous studies have also demonstrated genetic and biochemical interactions between Tup1p and class I histone deacetylases (HDACs) Rpd3p, Hos1p, Hos2p and the class II HDAC, Hda1p [29], [31], [32]. Deletion of many of these HDACs caused hyperacetylation of histones at promoter regions [29], [31], [33]. Tup1p-associated HDACs were found to be responsible for removal of the acetyl groups from the N-terminal tails of the core histones H2B, H3 and H4, indicating a reciprocal relationship between Tup1p binding and histone deacetylation. These findings suggested that Cyc8–Tup1 repression activity is modulated by changes in histone acetylation.

The yeast *FLO1* gene is regulated by the *TUP1* and *CYC8* (*SSN6*) gene products and is the dominant member of the *FLO* family of genes [34], [35]. *FLO1* gene expression causes flocculation which is the Ca^2 +^-dependent, nonsexual aggregation of yeast cells [36], [37], [38]. Flocculation has been shown to play a role in cellular resistance to external stresses such as heat, cold and various chemical reagents [39]. It is an important phenotype of brewing strains, but has become attenuated in some common laboratory strains [40]. We have previously shown that the Swi–Snf co-activator and the Cyc8–Tup1 complex influence nucleosomal arrays up to 5 kb upstream of the *FLO1* transcription start site. Thus, the chromatin in which the yeast *FLO1* promoter resides has a dynamic structure controlled by remodelling events which form the background in which promoter regulation takes place [41]. Hence, the *FLO1* gene represents a paradigm for chromatin-mediated regulation of gene transcription, and offers an amenable model system in which to investigate the mechanism of action of Cyc8–Tup1.

In this study, we propose that the *FLO1* gene represents a specific subset of genes subject to robust repression by Cyc8–Tup1 working cooperatively with the histone deacetylases, Hda1p and Rpd3p. We demonstrate that Cyc8–Tup1 localisation is focused at a hypersensitive site in the *FLO1* upstream nucleosome array. Removal of Cyc8–Tup1 leading to activation of the *FLO1* gene is coincident with long-range modulations of the histone H3 and H4 acetylation pattern and opening of the chromatin structure. Additionally, in the absence of Cyc8–Tup1, the occupancy of the HDACs, Rpd3p and Hda1p decreases in parallel with an increase in the occupancy of Swi–Snf at the site formerly occupied by Cyc8–Tup1. We show that Rpd3p and Hda1p are co-ordinately required for full repression but do not do so by enhancing Cyc8–Tup1 binding.

These data suggest that the long-range remodelling of the *FLO1* promoter and upstream chromatin requires distinctly bound Cyc8–Tup1and Swi–Snf and is mediated by histone acetylation.

## 2 Materials and methods

### 2.1 Yeast strains

*S. cerevisiae* strains were in the S288C background (Table S1) [42]. Yeast gene deletions and tagging were performed using PCR-based methods [43], [44]. All gene deletions were confirmed by PCR or Southern blot analysis of genomic DNA and assayed for appropriate phenotypes. PCR and Western blot analysis were used to confirm that the genomic copies of *CYC8*, *RPD3* and *HDA1* were correctly tagged with a C-terminal nine Myc epitope. Epitope tagged strains were assayed to confirm appropriate wild-type phenotypes. Cells were grown at 30 °C in YPD medium.

### 2.2 Chromatin immunoprecipitation

ChIP was performed as previously described [45], using the following antibodies: anti-acetyl-histone H3 lysine 9 (Millipore, 07-352); anti-acetyl-histone H4 (Millipore, 06-866); anti-histone H3 (Abcam, Ab1791) and anti-RNA Pol II (Covance, MMS-126R). The anti-Snf2p and anti-Tup1p antibodies were generous gifts from J. Reese. For the ChIP analysis of Myc-Rpd3p and Myc-Hda1p, cells were sequentially cross-linked with ethylene glycolbis[succinimidyl succinate] (EGS) and formaldehyde, as described [46]. The anti-cMyc antibody used was from Millipore (05-724). DNAs were analysed in triplicate by real-time quantitative PCR (qPCR) using a SYBR Green Master Mix (ABI) and ABI Step-One Plus PCR machine. The IP/input ratio for *FLO1* target sequences was normalised to the IP/input ratio at *TEL-VI* (Snf2p, Myc-Snf5p, H4ac, H3K9ac), *INT-V* (H3), or *STE6* (Tup1p, Myc-Cyc8p) sequences. The *STE6* site was used as a negative Cyc8–Tup1 binding site control. The *STE6* gene promoter is bound by Cyc8–Tup1 in *Mat*α cells, but is free of Cyc8–Tup1 in *Mat*a cells. All strains used in this study were *Mat*a. Primers used are listed in Table S2.

### 2.3 Chromatin analysis

Chromatin DNase I analysis was performed as previously described [47]. 200 μl nuclei were digested with 1, 5, 10 and 20 U of DNase I (Pharmacia) for 20 min at 37 °C (0.5 and 1 U for naked DNA). Indirect end-labelling mapping was performed as previously described [41], hybridising with probes − 3182 to − 2815 bp (*Hin*dIII) and − 2177 to − 1781 bp (*Bsr*BI). Chromatin restriction enzyme accessibility was assayed as previously described [48], hybridising with probes − 1283 to − 1054 bp (*Spe*I) and − 1670 to − 1878 bp (*Spe*I and *Rsa*I).

### 2.4 Protein analysis

Protein lysates were prepared using a TCA lysis buffer [49]. Lysates (20 μg) were electrophoresed on 8%–16% acrylamide-Tris HEPES gels (Pierce), and proteins were transferred to an Immobilon filter (Millipore; Billerica, MA). The filters were hybridised with H3 (Active Motif, 39163), H2A (Active Motif, 39235), H2B (Active Motif, 39237), H4 (Abcam, ab7311), Tup1p (J. Reese), Cyc8p (Santa Cruz,sc-11953), c-Myc (Millipore,05-724), or Beta actin (Abcam, ab8224) antibodies, and developed with ECL Western Blotting Substrate (Pierce).

### 2.5 Northern blot and RT-qPCR analysis

Northern analysis of mRNA expression was performed as previously described [50]. Total RNA was prepared, resolved on 1% denaturing agarose gels, transferred to Zeta-Probe GT membranes (Bio-Rad) and hybridised using DNA probes for *ACT1* and the complete *FLO1* coding region [41]. Band intensities were determined by phospho-imager analysis (FujiFilm FLA2000 FlouroImager). Where appropriate, *FLO1* values were normalised to *ACT1*. For RT-qPCR analysis of *FLO1* mRNA, RNA was extracted from cells by the hot phenol method [45], treated with DNase I (Promega) and used to generate cDNA with random primers and reverse transcriptase (Applied Biosystems). Negative controls with no reverse transcriptase were included. PCR reactions were performed in triplicate using a SYBR Green Master Mix (ABI) and ABI Step One Plus PCR machine. Values were normalised to *ACT1* RNA. Primers used are listed in Table S2.

### 2.6 Flocculation assay

Exponentially growing cells were resuspended to an equal cell density in YPD or YPD containing 100 mM EDTA (control). Equal volumes of cells were aliquoted into a tissue culture plate and agitated by shaking [51]. Five minutes after cessation of agitation, the plates were photographed. Cells displaying a flocculation phenotype aggregate in the absence of EDTA and are dispersed in the presence of EDTA (data not shown).

### 2.7 Analysis of Tup1p ChIP-seq, global transcription, Hda1 ChIP and nucleosome occupancy data

Expression array data generated by Chen et al. [24] were retrieved from ArrayExpress (E-GEOD-37466), RMA normalised, annotated for *S. cerevisiae* ORF and chromosomal locations (SGD), to calculate fold changes between wild-type and *TUP1* deletion strains. Tup1 protein binding sites were derived from ChIP-seq data generated by Wong and Struhl (NCBI accession no. SRA044839.1) using the algorithms and parameters described by the authors [23]. Peak maxima with p-value = 10^− 4^ (in one case compared with 10^− 2^) were assigned to the closest translational start codon (ATG) of annotated ORFs. Combination of ChIP and expression data, as well as plotting of graphs, was carried out in Excel using VBA custom scripts. For the Hda1p ChIP data analysis, lists of genes scoring above the False Discovery Rate (FDR) for each of three probe locations, as well as those shared between probes, were collated from Venters et al., 2011 [52]. These were subjected to further Venn analysis with gene subsets under study and plotted as a pie chart. Nucleosomal occupancy trace data sets were kindly provided from van Bakel et al., 2013 [53]. Using local R scripts, 1000 bp regions were retrieved from the genomic nucleosomal difference traces between a *tup1* mutant and wt for each subset of 90 locations, which were each plotted as an average trend over 1000 bp with standard deviation (SD). The SD for random regions (not shown for clarity) was very similar to the SD for non de-repressed genes.

## 3 Results

We previously reported that the subtelomeric *FLO1* gene promoter resides within a 5 kb chromatin region under the influence of long-range remodelling by the Cyc8–Tup1 and Swi–Snf complexes [41]. To gain more insight into this gene regulatory mechanism, we queried whether the *FLO1* gene was typical of a Cyc8–Tup1 repressed gene [23], [24].

### 3.1 Tup1p repression activity is enriched at chromosome subtelomeric regions

**Fig. 1:**
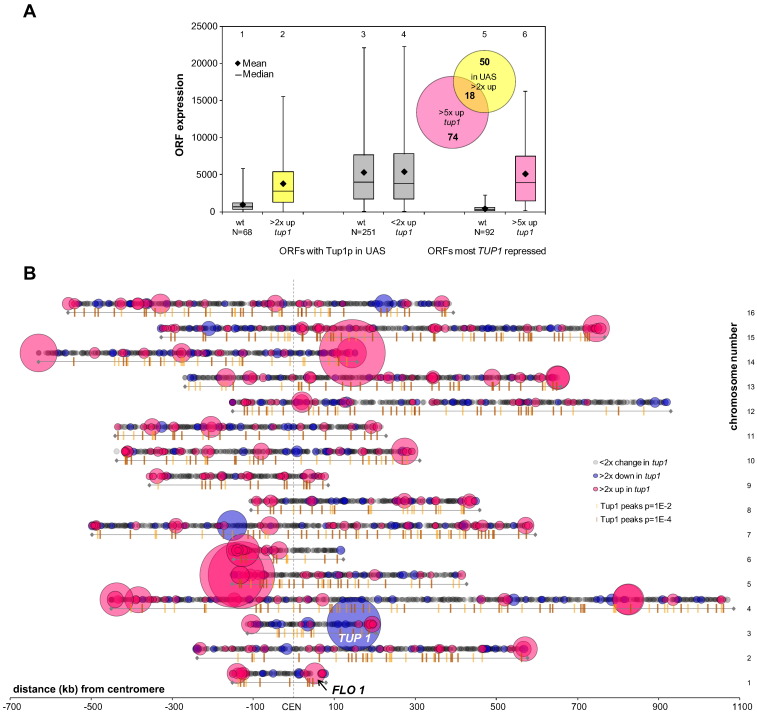
Tup1p repression activity is associated with subtelomeric regions. **(A)** Tup1p repression via the upstream activator sequence (UAS). Of 319 open reading frames (ORFs) with Tup1p occupancy less than 1000 bp upstream of the transcription start site (TSS), 68 ORFs are more than two-fold up-regulated in a *TUP1* deletion (*tup1*) strain (lanes 1–2). The remaining 251 ORFs are less than two-fold de-repressed when *TUP1* is deleted (lanes 3–4). The 92 ORFs de-repressed greater than 5-fold in a *tup1* mutant have very low wild-type expression (lanes 5–6). This set only partially overlaps with the promoter-bound Tup1p repressed subset (Venn diagram, inset). **(B)** Genes strongly repressed by Tup1p are enriched in subtelomeric regions. Tup1p occupancy across yeast chromosomes (ochre and brown bars represent Tup1p ChIP peaks) and ‘bubble’ plot showing the change in transcription in a *tup1* strain relative to wt. Circle area represents the fold change in transcription in the absence of Tup1p relative to wt levels, where grey, blue and pink circles represent < 2-fold change, > 2-fold repression and > 2-fold de-represssion, respectively.

We first compared global Tup1p occupancy data with the transcription profile of a *tup1* deletion strain to examine the correlation between Tup1p promoter occupancy and gene repression attributable to the *TUP1* gene [23], [24]. Our analysis revealed that of the 319 genes where a Tup1p peak could be located within 1000 bp upstream of the transcription start site, the deletion of *TUP1* resulted in less than two-fold increase in transcription of 251 (79%) of these genes (Fig. 1A, lanes 3 and 4). However, a subset of 68 (21%) of promoter-bound Tup1p genes is de-repressed more than two-fold in the absence of *TUP1*, while the low wild-type transcription levels of these genes indicate that they are indeed controlled by Tup1p repression (Fig. 1A, lanes 1 and 2). Furthermore, only 9 (3%) of the promoter-bound genes have less transcription in the *tup1* deletion strain, confirming the role of Tup1p as a repressor.

Conversely, analysis of the genes showing the greatest de-repression in the absence of *TUP1* (greater than five-fold de-repression) retrieved a set of 92 genes that were distinguished by very low levels of transcription in the wild-type strain (Fig. 1A, lanes 5 and 6). Interestingly, the majority of these genes most subject to Tup1p repression (74 genes) did not overlap with the promoter-bound Tup1p repressed subset of genes (Fig. 1A, Venn diagram, inset). Thus, those genes form a distinct subset under robust control of *TUP1*, which we suggest represents those genes directly switched on or off via the Cyc8–Tup1 complex.

We next compared the genome-wide Tup1p occupancy peak map with localised gene transcription changes in the absence of *TUP1* (Fig. 1B). The analysis revealed Tup1p occupancy (Fig. 1B, ticks) has a dissimilar distribution without strong bias towards the genes highly de-repressed when Tup1p is absent (Fig. 1B, pink circle areas). Interestingly, whereas Tup1p occupancy seems evenly distributed across the genome, many of the genes most strongly de-repressed in the absence of Tup1p are clustered within subtelomeric regions (Fig. 1B).

The *FLO1* gene resides within the subtelomeric region on the right arm of chromosome I and is representative of a set of subtelomeric genes that are highly de-repressed on a background of more moderately de-repressed genes when *TUP1* is deleted (Fig. 1B). The high levels of de-repression in the absence of Tup1p suggest that this subset of genes is predominantly repressed by Cyc8–Tup1 without possible compensatory repression mechanisms; in the absence of the co-repressor these genes are constitutively switched on. We believe that this set of highly de-repressed genes presents good models for clarifying current problems regarding the mechanism of Cyc8–Tup1 repression with fewer confounding factors. We have focused on the *FLO1* gene to address the relationship between Cyc8–Tup1, chromatin, gene repression and gene activation.

### 3.2 Cyc8–Tup1 is localised in the − 500 to − 1000 bp FLO1 upstream region and is required for repression of FLO1 gene transcription

**Fig. 2:**
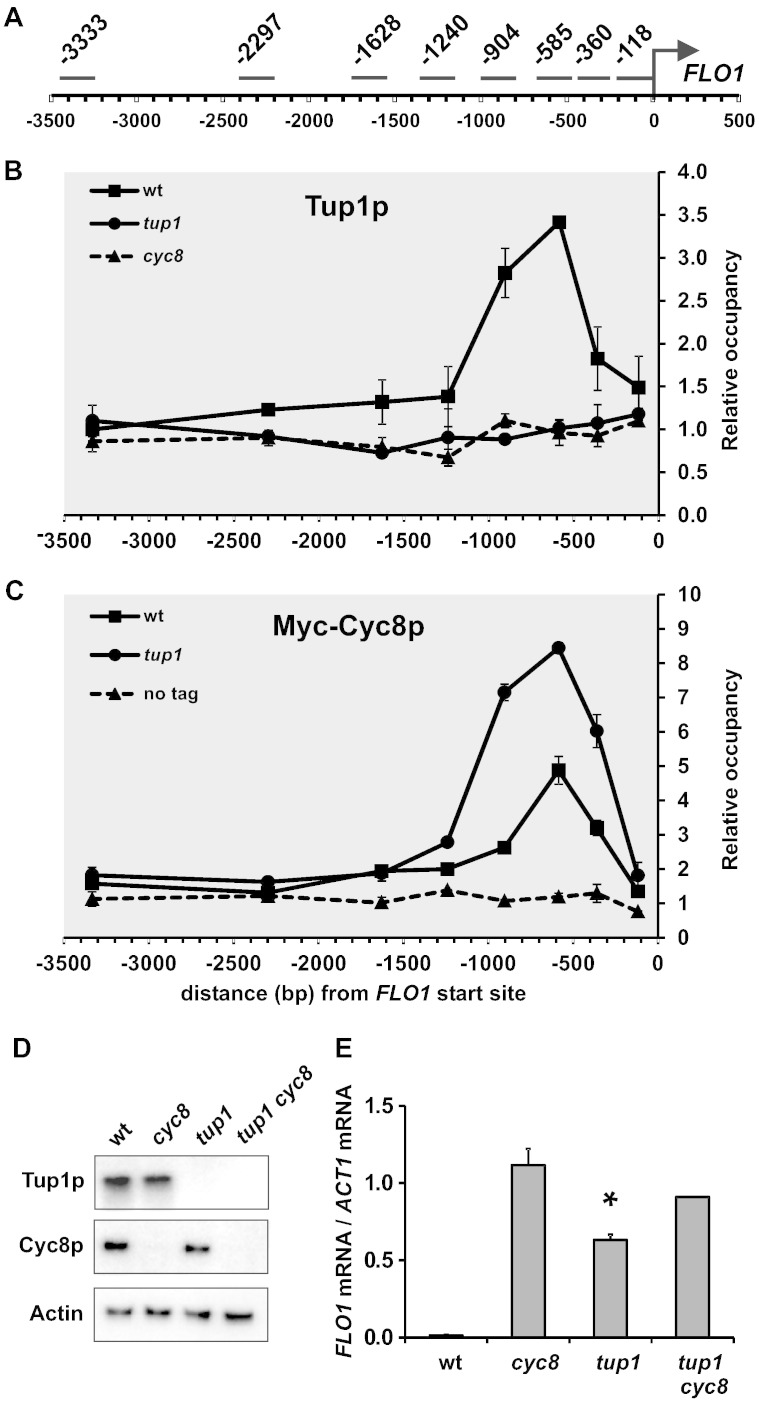
Tup1 and Cyc8 proteins are concentrated in a single location within 3.5 kb of the *FLO1* gene upstream region. (**A**) Diagram of the amplicons used in chromatin immunoprecipitation analysis covering a region up to 3500 base-pairs upstream (− 3500), labelled by the distance (bp) from their midpoints to the *FLO1* translation start site (+ 1). Cross-linked chromatin fragments from wild-type (wt), *CYC8* and *TUP1* deleted strains (*cyc8* and *tup1* respectively) were immunoprecipitated with antibodies against Tup1p (**B**) and Myc-Cyc8p (**C**) and the DNA content analysed by qPCR. Occupancies were normalised to the *STE6* gene promoter. The results represent the average from two to three independent experiments with bars representing SEM. (**D**) Western blot analysis of Tup1p and Cyc8p in wt, *CYC8* and *TUP1* single and double deleted strains (wt, *cyc8*, *tup1* and *tup1 cyc8* respectively). Representative results from two to three independent experiments are shown. (**E**) *FLO1* transcript levels relative to *ACT1* mRNA levels were measured in wt, *cyc8, tup1* and *tup1 cyc8* mutant strains by RT-qPCR and normalised to wt. The results represent the average from three independent experiments, with bars representing SEM. The asterisk indicates a statistically significant difference between the *tup1* and *cyc8* mutants as determined by the Student’s t test (p < 0.05).

To investigate the molecular mechanism of *FLO1* gene repression by the Cyc8–Tup1 complex, we mapped Tup1p and Cyc8p localisation over a 3.5 kb chromatin region upstream of the *FLO1* coding sequence (Fig. 2A). The results confirmed that Tup1p and Cyc8p were concentrated in the upstream region between − 500 and − 1000 bp in wild-type (wt) strains (Fig. 2B and C, wt) [23], [54], [55]. To test the contribution of the individual Cyc8 and Tup1 protein subunits to localisation of the complex, we constructed *CYC8* and *TUP1* deletion mutants (*cyc8* and *tup1*) and measured the respective Tup1p and Cyc8p abundance and occupancies.

Western blot analysis of Tup1p and Cyc8p levels in the respective *cyc8* and *tup1* deletion mutants confirmed the stability of each subunit was unaffected by the absence of the other (Fig. 2D and Supplemental Fig. S1). ChIP analysis revealed that the peak of Tup1p around − 700 bp upstream of *FLO1* in wt strains was completely absent in *cyc8* strains (Fig. 2B, *cyc8*). This suggests Tup1p is recruited in the context of the Cyc8–Tup1 complex confirming observations at the *RNR2* and *STE6* genes [30]. Surprisingly, the peak of Cyc8p was significantly increased in *tup1* strains. Thus, Cyc8p remains bound to the *FLO1* promoter in the absence of Tup1p where it either increases in occupancy or is more amenable to ChIP analysis.

We next confirmed the role of Cyc8–Tup1 in *FLO1* repression by measuring *FLO1* transcription in *cyc8* and *tup1* single and double deletion mutant strains. The data revealed that de-repression of *FLO1* transcription was greatest in *cyc8* strains compared to *tup1* strains and wt (Fig. 2E). Analysis in a *tup1 cyc8* double mutant showed that *FLO1* de-repression was similar to that seen in the *cyc8* single mutant. Taken together, the results suggest that Cyc8p occupancy in the absence of Tup1p either directly or indirectly imparts partial *FLO1* gene repression. We conclude that the *cyc8* mutant, and not the *tup1* mutant, is representative of a strain defective for Cyc8–Tup1 complex binding and activity at *FLO1*. Furthermore, Tup1p is representative of Cyc8–Tup1 complex occupancy at *FLO1*.

### 3.3 Cyc8–Tup1 localises within a DNase I hypersensitive site and is required for chromatin organisation at the repressed FLO1 promoter and upstream region

**Fig. 3:**
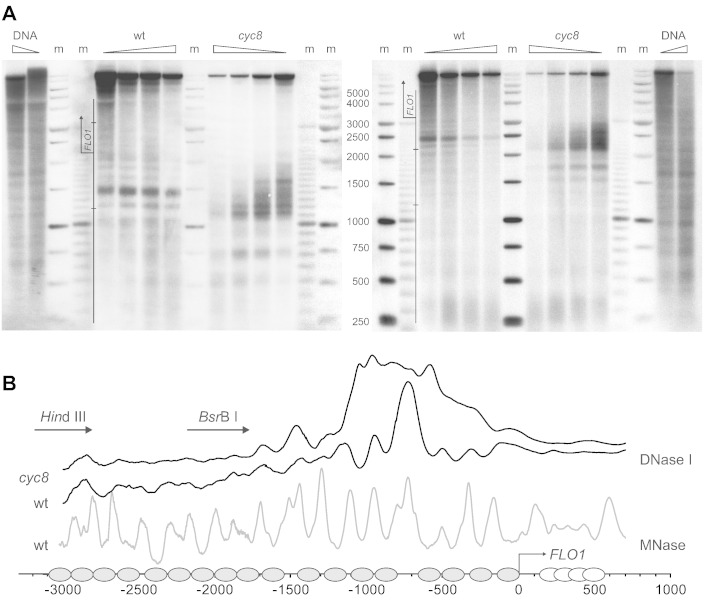
A DNase I hypersensitive site overlaps with the binding site of Cyc8–Tup1 and greatly expands in its absence. Indirect end-labelling mapping of the DNase I cleavage pattern of chromatin in the *FLO1* upstream region. (**A**) Southern blots of DNA agarose gel separation of DNase I digestions of wt and *cyc8* chromatin and naked DNA, probed relative to restriction sites *Bsr*B I at − 2193 bp (left) and *Hin*dIII at − 3185 bp (right). (**B**) Composite map of gel intensity traces of A (shown for chromatin only; black traces) recalculated to linear bp scale using the equation of the DNA standard curve determined by polynomial regression analysis of the markers. Hybridisation probes used in A are indicated by arrows. Also shown is the wt chromatin MNase pattern (grey trace), which matches the DNase I pattern, and the putative positions of nucleosomes in this region [41].

Cyc8–Tup1 has been proposed to promote gene repression by stabilising promoter nucleosomes to form repressive chromatin structures [41], [50], [55], [56], [57], [58]. We therefore analysed the DNase I sensitivity in yeast nuclei over a distance of 3 kb of upstream chromatin and 1.5 kb of *FLO1* coding region (not shown), using the indirect end-labelling technique. Fig. 3A shows the digestion patterns from the vantage point of two restriction sites in the upstream region. We discovered a striking DNase I hypersensitive site around − 700 bp upstream of *FLO1* in repressed wild-type chromatin [Fig. 3B, wt (black gel trace)]. This single hypersensitive site of about 100 bp wide coincides with the focus of Tup1p and Cyc8p ChIP localisation. Our previous micrococcal nuclease (MNase) mapping of the nucleosome positions within the upstream chromatin had indicated a very long ‘linker’ between nucleosomes in this area [Fig. 3B, wt (grey gel trace)] [41]. We conclude that Cyc8–Tup1 is recruited to a distinct opening within the nucleosomal array. In the immediate vicinity of this opening, DNase I cuts a nucleosomal ladder pattern, indicative of a well-spaced ordered array of nucleosomes (Fig. 3A and B). In contrast, the chromatin further upstream appears more inaccessible to DNase I, which could be explained by sterical hindrance from close spacing of nucleosomes [59].

Interestingly, when Cyc8–Tup1 repression of *FLO1* is relieved in *cyc8* strains, the DNase I digestion pattern shows a dramatic expansion of the hypersensitive site in the chromatin, which now covers a space several nucleosomes wide (Fig. 3A and B, *cyc8*). Again, DNase I cuts a nucleosomal ladder pattern immediately upstream of this widened hypersensitive region, demonstrating further opening of the adjacent chromatin structure. This result is consistent with our previous micrococcal nuclease analysis, which showed a gross disruption of the nucleosomal array in this region flanked by more widely spaced nucleosomes [41]. These data suggest a role for Cyc8–Tup1 in the organisation of repressive regions of chromatin or in antagonising chromatin disruption [23], [41], [50], [55].

### 3.4 Histone H3 occupancy at the FLO1 promoter and upstream region is reduced in the absence of Cyc8–Tup1

**Fig. 4:**
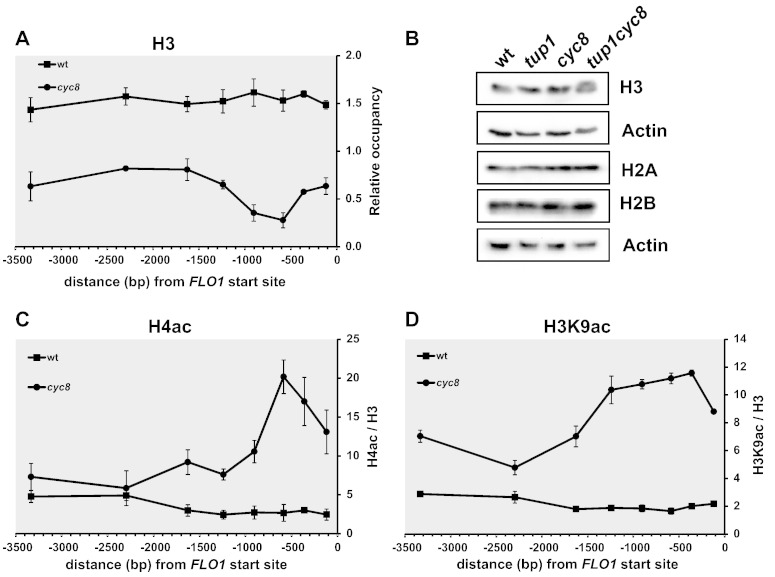
(**A**) Histone H3 occupancy patterns are suggestive of a lower nucleosome density over the disrupted *FLO1* proximal promoter and upstream region in *cyc8* strains. ChIP analysis using antibodies against histone H3. Graph plotting histone H3 occupancy at the *FLO1* promoter and upstream region in wt and *cyc8* deletion strains. Histone H3 occupancies were normalised to the ORF-free region, *INT-V*. The amplicon positions are as described in Fig. 2A. The results represent means from three to four independent experiments, with bars depicting SEM. (**B**) Western blot analysis of histone levels in wt, *CYC8* and *TUP1* deleted strains. (**C and D**) In the absence of Cyc8–Tup1, levels of histone H4 and H3 acetylation increase in adjacent regions. The level of acetylated histones H4 (**C**), and H3 (**D**), over the *FLO1* promoter and upstream region were determined by ChIP using antibodies against acetylated histone H4 lysines 5, 8, 12 and 16 (H4Ac), and acetylated histone H3 lysine 9 (H3K9ac), respectively. H4Ac and H3K9ac levels in wt and *cyc8* deletion strains were normalised to *TEL-VI* and are shown relative to histone H3 levels. The amplicon positions are as described in Fig. 2A. The results represent the mean from three to four independent experiments, with bars depicting SEM.

The indirect end-labelling analysis experiment indicates the gross disruption of the nucleosomal array at the *FLO1* promoter region when Cyc8–Tup1 repression is relieved. However, the severe disruption in the micrococcal nuclease pattern corresponding to the expansive DNase I hypersensitive site was not accompanied by a reversal to the naked DNA digestion pattern [41]. Furthermore, restriction enzyme accessibility assay results were not consistent with digestion of naked DNA (Supplemental Fig. S2), suggesting the remodelled region was not completely devoid of nucleosomes. To directly determine the fate of nucleosomes at the de-repressed *FLO1* promoter and upstream region, we examined histone H3 occupancy in *cyc8* mutant strains by ChIP (Fig. 4A).

In wild-type cells, where *FLO1* transcription is repressed, histone H3 occupancy at the *FLO1* promoter and upstream region was higher than at the control intergenic region to which the H3 data were normalised (Fig. 4A, wt). This suggests that there is a relatively high histone density at *FLO1* compared to the control non-transcribed region considered representative of the global chromatin background. Strikingly, in the absence of Cyc8p, when *FLO1* transcription is de-repressed, there is a general decrease in histone H3 occupancy across the entire region tested (Fig. 4A, *cyc8*). However, the most dramatic histone density decrease was in the region adjacent to the site previously bound by Cyc8–Tup1, around − 0.5 kb to − 1 kb upstream of the *FLO1* transcription start site. Western blot analysis shows that histone levels in the *cyc8* mutant are the same as wt suggesting that the dramatic histone depletion is specific to the *FLO1* promoter region and not due to a general loss of histones in this mutant (Fig. 4B).

The results over the proximal promoter region are consistent with our previous finding that the nucleosome array in the *FLO1* promoter region is severely disrupted in *cyc8* strains with a likely loss of nucleosome density, rather than complete nucleosome removal, due to histone eviction [41]. Overall, the data suggest that Cyc8–Tup1 acts to establish or maintain a highly organised chromatin structure over the repressed wild-type *FLO1* gene promoter and upstream region.

### 3.5 Cyc8–Tup1 is required for histone H3 and H4 deacetylation at the FLO1 promoter region

The association of Cyc8–Tup1 with chromatin *in vivo* has been linked to low levels of histone acetylation [28], [29], [30]. We anticipated that in wild-type strains, the histone tails co-localising in the chromatin with Cyc8–Tup1 in the *FLO1* promoter region would be hypoacetylated. We therefore performed ChIP to analyse histone acetylation in this region by using antibodies against acetylated histone H3 (lysine 9), acetylated histone H4 (lysine 12, not shown), or all acetylated isoforms of histone H4 (lysines 5, 8, 12 and 16). At each position tested, histone acetylation levels were normalised to the respective histone H3 occupancy at that site (Fig. 4C and D).

In the wild-type strain, where *FLO1* transcription is repressed, levels of acetylated histone H4 (H4ac) and acetylated lysine 9 of histone H3 (H3K9ac) were low over the entire 3.5 kb *FLO1* promoter and upstream region (Fig. 4C and D, wt). Conversely, in *cyc8* strains where *FLO1* transcription is de-repressed, increased H4ac between − 2 kb and the *FLO1* transcription start site was evident, with a sharp peak of increased acetylation present in the − 0.6 kb to − 0.4 kb area immediately adjacent to where Tup1p localises in wild-type strains (Fig. 4C, *cyc8*). Deletion of *CYC8* resulted in lysine 9 of histone H3 being broadly hyperacetylated over the entire 3.5 kb *FLO1* upstream region, but with the greatest increase in acetylation levels again evident across the − 1.5 kb upstream *FLO1* region (Fig. 4D, *cyc8*). These results suggest a role for Cyc8–Tup1 in either promoting histone deacetylation or blocking histone acetylation across the region extending up to − 3.5 kb upstream of the *FLO1* transcription start site.

### 3.6 Disruption of the HDA1 and RPD3 histone deacetylase genes results in partial de-repression of FLO1 gene transcription

Previous reports have connected Cyc8–Tup1 to class I histone deacetylases (HDACs) Rpd3p and Hos2p [31], and Tup1p has been linked to the class II HDAC Hda1p [33]. Flocculation was also reported to be induced during high-gravity fermentation in an *hda1* mutant [60]. In order to identify which HDACs, if any, were involved in *FLO1* gene repression in our system, mutations in various HDACs were prepared and screened for *FLO1* transcription. If the deletion of an HDAC gave rise to *FLO1* transcription it could be inferred to play a role in *FLO1* gene repression. We therefore made deletions of class I, II and III HDAC genes either individually, or in combination, and assayed for flocculation and *FLO1* transcription.

**Fig. 5:**
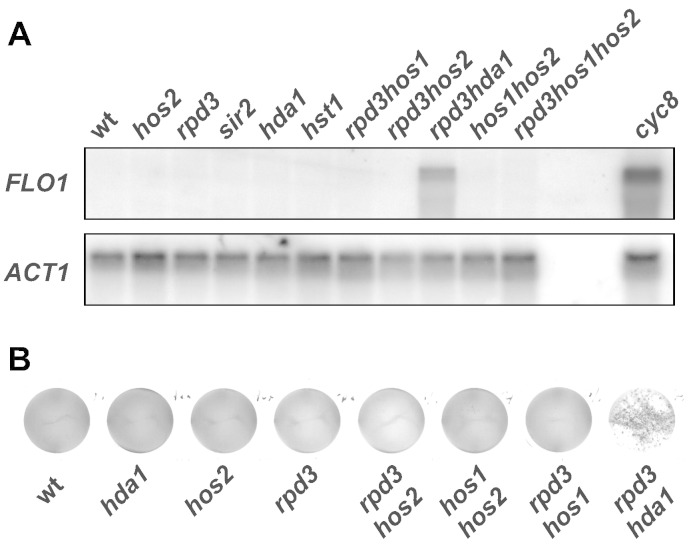
HDAC genes *HDA1* and *RPD3* are involved in repression of *FLO1* gene transcription. (**A**) Northern blot analysis of *FLO1* mRNA in wt and strains deleted for the HDAC genes indicated. The blot was re-probed with *ACT1* as a loading control. Two lanes between the *rpd3 hos1 hos2* and *cyc8* samples were left empty to prevent ‘bleed’ on the blot from the high signal expected in the *cyc8* sample. (**B**) Analysis of flocculation phenotype in wt and strains deleted for the HDAC genes indicated. Flocculation was assayed by photographing yeast cells in liquid culture five minutes after cessation of agitation.

As shown in Fig. 5A, single gene deletions of various HDAC-encoding genes did not de-repress *FLO1* transcription. However, a double deletion of both the *HDA1* and *RPD3* HDAC-encoding genes caused a partial de-repression of *FLO1* transcription. Indeed, *FLO1* de-repression in the absence of both Hda1p and Rpd3p was measured at 21% of that detected in the absence of *CYC8*. The resultant flocculation phenotype exhibited by the *rpd3 hda1* double deletion mutant cells is shown in Fig. 5B. These data suggest the class I and II HDACs, Hda1p and Rpd3p respectively, both contribute to repression of *FLO1* gene transcription.

### 3.7 Disruption of HDA1 and RPD3 results in reduced histone H3 occupancy at the FLO1 promoter

**Fig. 6:**
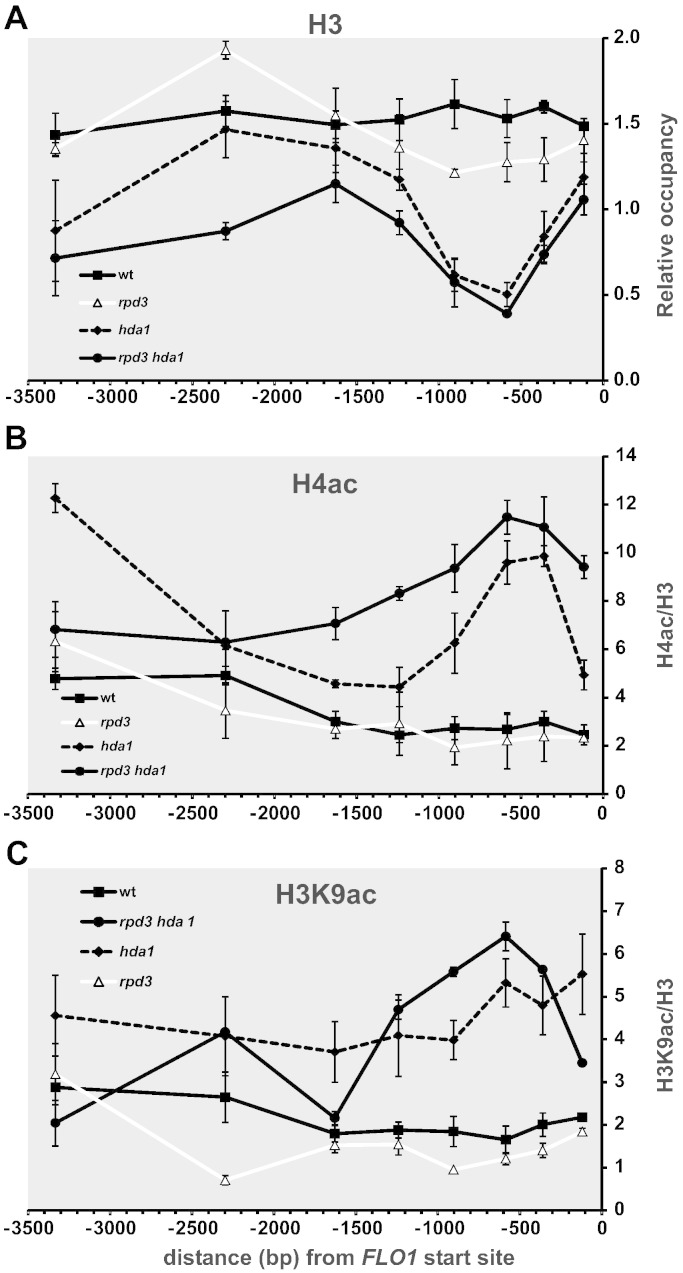
Histone occupancy and histone acetylation are altered in an *rpd3 hda1* double mutant. (**A**) Histone occupancy is reduced in the absence of Rpd3p and Hda1p. ChIP analysis using antibodies against histone H3. Graph plotting histone H3 occupancy at the *FLO1* promoter and upstream region in wt, *rpd3, hda1* and *rpd3 hda1* deletion strains. Histone H3 occupancies were normalised to the ORF-free region, *INT-V*. The results represent means from three to four independent experiments, with bars depicting SEM. The wt data shown were to aid comparison, and are the same data as shown in Fig. 4A. (**B and C**) Histone H4 and H3 acetylation levels increase across the *FLO1* promoter and upstream region in the absence of Hda1p and Rpd3p. The level of acetylated histones H4 (**B**) and H3 (**C**) over the *FLO1* promoter and upstream region in wt, *rpd3*, *hda1* and *rpd3 hda1* deletion strains was determined by ChIP as described in Fig. 4. H4ac and H3K9ac occupancy was normalised to histone H3 levels. The results represent the mean from three to four independent experiments, with bars depicting SEM. The wt data were to aid comparison, and are the same data as shown in Figs. 4C and D. (**A–C**) The amplicon positions are as described in Fig. 2A.

The de-repression of *FLO1* transcription in the absence of Cyc8–Tup1 coincides with a dramatic reduction of histone occupancy across the *FLO1* promoter and upstream region, potentially as a result of histone eviction (Fig. 4A, *cyc8*). We therefore analysed histone occupancy in the *rpd3 hda1* double deletion mutant compared to *rpd3*, *hda1*, *cyc8* single mutants and wild-type, to determine if a histone occupancy reduction was associated with the partial levels of *FLO1* de-repression detected in the *rpd3 hda1* double mutant (Fig. 6A).

Using ChIP we could detect no difference in histone H3 levels in the *rpd3* single mutant compared to wild-type, which correlated with the absence of *FLO1* transcription in this strain (Fig. 6A, *rpd3*). Surprisingly, histones were found to be depleted from the − 0.5 to – 1.0 kb *FLO1* promoter region in the *hda1* single mutant, despite there being no detectable *FLO1* transcriptional de-repression in this strain (Fig. 6A, *hda1*). In the absence of both the *RPD3* and *HDA1* genes however, where *FLO1* is partially de-repressed, there was a similar decrease in histone occupancy at the *FLO1* proximal promoter region as seen in the *hda1* single mutant, but the decrease in histone occupancy extended further to cover the − 2.5 kb *FLO1* upstream region (Fig. 6A, compare *rpd3 hda1* and *hda1*). Importantly, the loss of histones in the *rpd3 hda1* double mutant was not as dramatic as the histone depletion evident in the absence of Cyc8p where maximal *FLO1* de-repression occurs (compare Fig. 6A, *rpd3 hda1* and Fig. 4A, *cyc8*). Overall, the data suggest that both Hda1p and Rpd3p are required for the establishment or maintenance of histone occupancy at the repressed *FLO1* gene.

### 3.8 Disruption of HDA1 and RPD3 results in histone H3 and H4 hyperacetylation at the FLO1 promoter

If the role of Rpd3p and Hda1p in *FLO1* repression is to deacetylate histones at the *FLO1* promoter and upstream region, it would be predicted that the histones in this region would be hyperacetylated in the absence of both Hda1p and Rpd3p. We therefore analysed histone H3 lysine 9 and histone H4 acetylation levels in *hda1*, *rpd3* and *rpd3 hda1* mutants compared to wild-type by ChIP. Histone acetylation levels were normalised to histone levels in each strain to account for differences in histone occupancy (Fig. 6B and C). In the *rpd3* mutant we could not discern any changes in histone H4 or H3 acetylation levels compared to wild-type over the entire *FLO1* upstream region tested (Fig. 6B and C, *rpd3*). In contrast, in *hda1* cells, we observed a distinct increase in H4 acetylation levels focussed within the − 1 kb *FLO1* promoter proximal region (Fig. 6B, *hda1*). Additionally, the pattern of H3K9ac in *hda1* cells showed an increase that was consistently higher than the wild-type profile across the 2 kb *FLO1* upstream region (Fig. 6C, *hda1*). It is important to note that neither single *RPD3* nor *HDA1* deletion de-represses the *FLO1* gene, as shown by lack of a flocculation phenotype and the absence of signal in Northern blots as well as microarray data [61], [62].

However, when both the *HDA1* and *RPD3* genes were deleted, a strain in which *FLO1* transcription is partially de-repressed, the pattern of H4 acetylation was generally higher and broader than in the *hda1* single mutant, encompassing almost 2 kb of the *FLO1* upstream region (Fig. 6B, *rpd3 hda1*). Histone H3K9ac levels in the *rpd3 hda1* double deletion mutant were also reproducibly higher than the acetylation levels observed in the *hda1* single mutant, with the greatest increase in the region between − 1.0 kb and − 0.3 kb upstream of the *FLO1* transcription start site (Fig. 6C, *rpd3 hda1*). These data suggest that there is a biased redundancy between Hda1p and Rpd3p histone deacetylation activities at the wild-type *FLO1* promoter and upstream region which contributes to histone deacetylation and gene repression: Hda1p can compensate for the absence of Rpd3p deacetylase activity, whereas Rpd3p may only partially compensate for loss of Hda1p.

Importantly, histone H4 and H3 acetylation levels at the *FLO1* upstream region in the *rpd3 hda1* double mutant were lower than those in the absence of either Tup1p or Cyc8p, but showed a similar profile (compare *rpd3 hda1* in Fig. 6B and C with Fig. 4C and D, *cyc8*). This result correlates with the lower level of *FLO1* de-repression in the *rpd3 hda1* mutant compared to the *cyc8* mutant. These data do not exclude that other HDACs may also contribute to *FLO1* promoter deacetylation and gene repression. However, the absence of previously reported Cyc8–Tup1 associated HDACs Hos1p and Hos2p [31] had no effect on *FLO1* transcription, either alone or in combination with an *rpd3* deletion (Fig. 5A and data not shown).

### 3.9 Hda1p and Rpd3p bind the repressed FLO1 promoter and upstream region

**Fig. 7:**
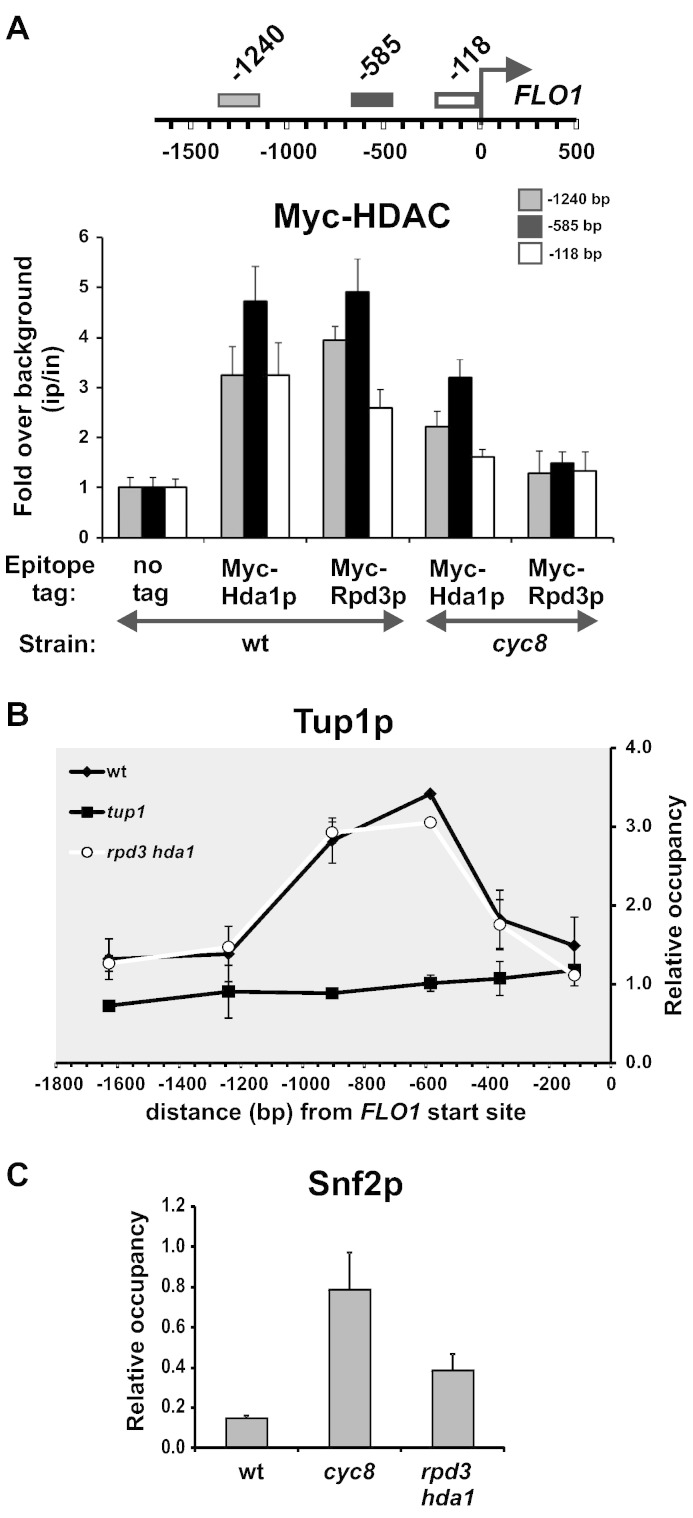
The absence of Cyc8–Tup1 correlates with decreased HDAC occupancy and increased Swi–Snf co-activator occupancy. (**A**) Hda1p and Rpd3p are present at the *FLO1* promoter, but their occupancy is reduced in the absence of *CYC8*. ChIP analysis of Myc-Hda1p and Myc-Rpd3p association with the *FLO1* promoter in wt and *cyc8* strains. Occupancies were normalised to the untagged control. (**B**) Tup1p occupancy is not altered in the absence of *RPD3* and *HDA1*. Tup1p ChIP at the *FLO1* promoter and upstream region. Occupancies were normalised to the *STE6* promoter. The wt and *tup1* data shown were to aid comparison, and are the same data as shown in Fig. 2B. (**C**) Snf2p co-activator binds the *FLO1* promoter in the absence of *CYC8* or *RPD3* and *HDA1*. Snf2p ChIP analysis at the − 585 bp *FLO1* promoter region in wt, *cyc8* and *rpd3 hda1* strains. Snf2p occupancy was normalised to the telomeric region, *TEL-VI*. (**A–C**) The results represent the mean from three to four independent experiments, with bars depicting SEM.

Our results suggest that Hda1p and Rpd3p histone deacetylase activities contribute to histone deacetylation at the *FLO1* promoter and upstream region and are required for *FLO1* gene repression. To test if this effect was direct, we used ChIP to determine if we could detect Hda1p and Rpd3p occupancy at the repressed *FLO1* promoter and upstream region. In the wild-type strain, the occupancy of both epitope-tagged Hda1p and Rpd3p HDACs was confirmed across the repressed *FLO1* promoter proximal region (Fig. 7A, wt and Supplementary Fig. S3). Interestingly, the data showed a reproducible enrichment of both HDACs centred at the site of Cyc8–Tup1 occupancy at − 585 bp proximal to the *FLO1* start site. This finding confirms that both Hda1p and Rpd3p are bound at the repressed *FLO1* promoter.

### 3.10 Regulatory interplay between Cyc8–Tup1 and Hda1p and Rpd3p

One model for the activity of the Cyc8–Tup1 complex is that it recruits HDACs to deacetylate histones at target promoters. The resultant histone deacetylation has been proposed to promote further recruitment of Cyc8–Tup1 thereby reinforcing its occupancy to strengthen gene repression [7], [31]. Considering our findings of a potential overlap in the sites of occupancy between Cyc8–Tup1 and Rpd3p and Hda1p at the *FLO1* promoter, we investigated if there was evidence to support the proposed regulatory interplay between the HDACs and Cyc8–Tup1 at *FLO1*. The model would predict that HDAC occupancy would be reduced in the absence of Cyc8–Tup1, and that Cyc8–Tup1 occupancy would be reduced in the absence of the HDACs. We first examined Hda1p and Rpd3p occupancy at *FLO1* in the absence of *CYC8*. Fig. 7A shows that the occupancy of both Hda1p and Rpd3p was reduced in a *cyc8* mutant. This result supports a role for Cyc8–Tup1 in promoting HDAC occupancy at the repressed *FLO1* promoter.

Next, we determined if the HDACs Hda1p and Rpd3p modulated the binding of Tup1p at the *FLO1* promoter. Using ChIP, we measured the occupancy of Tup1p in the *rpd3 hda1* double mutant where we had previously shown histone hyperacetylation across the *FLO1* promoter region and partial *FLO1* de-repression. However, no difference in Tup1p occupancy at the *FLO1* promoter region was detected (Fig. 7B, compare *rpd3 hda1* and wt). Thus, the data indicate that Cyc8–Tup1 regulates the occupancy of Hda1p and Rpd3p at the repressed *FLO1* promoter, however Hda1p and Rpd3p and their associated HDAC activities, do not regulate the level of Cyc8–Tup1 occupancy. The data also demonstrate that *FLO1* transcription can occur in the presence of Cyc8–Tup1.

### 3.11 Snf2p binds the de-repressed FLO1 promoter

We have previously shown that in the absence of Cyc8–Tup1, *FLO1* transcription and the concomitant promoter and upstream region nucleosome rearrangements are dependent upon the Swi–Snf complex. [41] We therefore determined if Swi–Snf was enriched at the *FLO1* promoter in the absence of Cyc8p when transcription is de-repressed. Using ChIP analysis we measured the occupancy of the Snf2p subunit of Swi–Snf at the *FLO1* promoter region in the repressed wild-type strain, and in the *cyc8* and *rpd3 hda1* mutants. The results showed that Snf2p occupancy was enriched at the *FLO1* promoter in the *cyc8* mutant strain compared to wild-type (Fig. 7C and Supplemental Fig. S4). Furthermore, Snf2p occupancy was enriched relative to wild-type levels in the *rpd3 hda1*double mutant, but was present at lower levels than that detected in the *cyc8* mutant. Interestingly, Snf2p was recruited to the same region previously occupied by Cyc8–Tup1 in the *cyc8* mutant, whilst in the *rpd3 hda1* mutant Snf2p potentially co-localised with fully bound Cyc8–Tup1. Thus, Snf2p occupancy at the *FLO1* gene promoter correlates with the level of *FLO1* transcription, and shows the greatest binding in the absence of Cyc8–Tup1 at its former binding site.

### 3.12 The FLO1 gene is representative of genes that are strongly de-repressed in the absence of TUP1

**Fig. 8:**
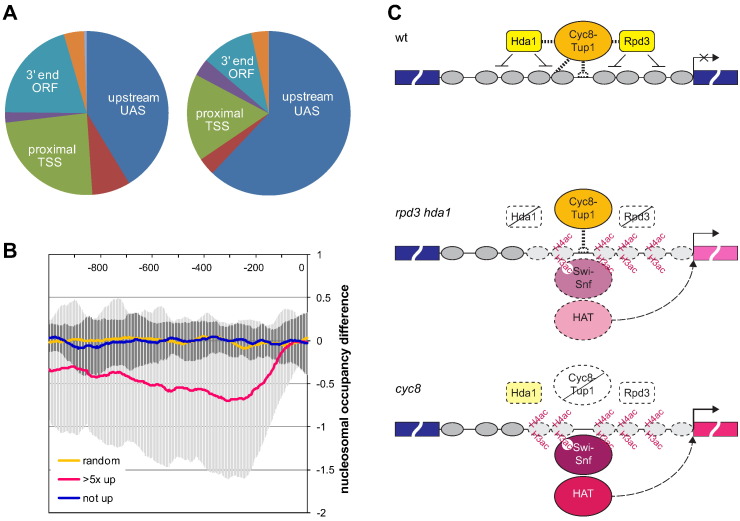
(**A**) *TUP1* repressed upstream regions associate with UAS centred Hda1p. Relative distribution of Hda1p over gene regulatory regions probed by ChIP in wt strains, charted for whole genome (left), and the gene set > 5 × de-repressed in a *tup1* mutant (N = 90, right). Hda1p presence over the − 320 to − 260 upstream region (‘UAS’) is 81% increased in this set (p = 0.005) compared with whole genome, while it is lower in the − 90 to − 30 proximal region (‘TSS’) and the 3’ end ‘ORF’ region. ORFs scoring in two regions are plotted in the areas between major categories. Only ORFs scoring above the FDR are included in the pie chart, while detectable Hda1p was 20% increased overall (p = 0.05). (**B**) *TUP1* de-repressed upstream regions have a high incidence of nucleosome depletion. Average of nucleosomal occupancy differences between *tup1* and wt strains is plotted over the upstream 1000 bp region. Genes > 5 × de-repressed (N = 90) in a *tup1* mutant show a lower average trace than non de-repressed genes (N = 90), or a set of random genomic 1000 bp regions (N = 90). Neither control shows significant changes in nucleosome occupancy between wt and mutant. De-repressed gene changes (*tup1*) show a wide variance ranging from wt occupancy to depletion occurring with high incidence across a wide upstream region (error bars, 1 SD). (**C**) Model for the role of Cyc8–Tup1 in *FLO1* gene repression. In wt, Cyc8–Tup1 binds to a distinct site at the *FLO1* promoter and is associated with an array of strongly positioned, deacetylated nucleosomes covering the promoter and upstream region. The histone deacetylases (HDACs), Rpd3p and Hda1p bind the promoter in a Cyc8–Tup1 dependent manner and contribute to nucleosome positioning, histone deacetylation and gene repression. In the absence of Cyc8–Tup1 (*cyc8* mutant), there is a gross remodelling of *FLO1* promoter and upstream chromatin involving nucleosome acetylation, rearrangement and eviction which accompanies *FLO1* gene de-repression. The occupancy of Rpd3p and Hda1p (to a lesser extent) is reduced. Swi–Snf is recruited to the site previously occupied by Cyc8–Tup1 and potentially directs nucleosome disruption. The increased chromatin acetylation in the *cyc8* mutant suggests that histone acetyltransferase (HAT) recruitment and activity occur in this region. In the absence of the HDACs Rpd3p and Hda1p (*rpd3 hda1* double mutant), Tup1p occupancy persists. Swi–Snf occupancy, nucleosome rearrangement and eviction also occur at the *FLO1* promoter and upstream chromatin but at a reduced level compared to *cyc8*, and *FLO1* is only partially de-repressed (compared to *cyc8*). Histone acetylation over the region is also lower than in the *cyc8* mutant suggesting decreased HAT occupancy or activity in this strain.

The pivotal role that *HDA1* plays in repression at *FLO1* (Fig. 5, Fig. 6, Fig. 7) raises the question of whether this mechanism is employed at other gene promoters. A yeast genome-wide study of the regulator co-occupancy network identified a ‘lowly transcribed’ cluster of genes that contained generally repressive chromatin remodellers and histone deacetylases, such as Isw1p, Isw2p, Cyc8p/Ssn6p, Hos1p, and Hda1p [52]. This study assessed binding between distal versus proximal promoter regions as well as the 3’ ends of genes by ChIP. *HDA1* has widespread functions in the yeast genome [52], [63]. We therefore asked whether the set of genes that is strongly de-repressed in the absence of *TUP1* has a specific Hda1p binding profile compared with the whole genome Hda1p binding profile. Analysis of Hda1p ChIP data over the three sites demonstrated an 81% enrichment (p = 0.005) of Hda1p on the more distal − 320 to − 260 upstream activator sequence (UAS) site for this set of genes, while 20% more Hda1p binding was detected overall (Fig. 8A). This significantly different upstream region binding profile of Hda1p compared with whole genome is consistent with its specific involvement at this set of genes. Furthermore, Rpd3p, Cyc8-Tup1, and Swi–Snf were amongst factors found to preferentially occupy this upstream promoter region [52].

The extensive nucleosome depletion observed at *FLO1* in the absence of *TUP1* (Fig. 3, Fig. 4) additionally poses the question of whether this is also part of a mechanism that occurs at other promoters. A compendium study assessed the respective roles of a large collection of yeast genes in genome-wide nucleosome positioning and transcription in loss of function gene mutants. This study identified the *tup1* and *cyc8* single deletion mutants as having a particularly strong relationship between changes in nucleosome occupancy in the − 200 to transcription start site (TSS) region and the expression of their target genes [53]. We therefore analysed data for the − 1000 bp to TSS region, where nucleosome depletion is observed at the de-repressed *FLO1* gene, for changes in nucleosome occupancy in the set of genes that is strongly de-repressed in the absence of *TUP1*. The set shows a high incidence of negative nucleosome occupancy changes in mutant chromatin compared with wild-type, resulting in a significantly lower average trace compared with the average for non de-repressed genes, or for random regions of the genome, respectively (Fig. 8B). These control sets showed no nucleosomal occupancy change overall, suggesting that nucleosome depletion is specific to this de-repressed set of genes. Nucleosome loss at individual genes occurs at various locations over this upstream region, resulting in a wide variance in occupancy around the calculated lower average at any particular position. These dramatic changes in occupancy are not commonly observed for single chromatin mutants, as they may only manifest their effects under conditions of stress, or may be subject to inherent redundancy of factors involved in chromatin homeostasis [53]. The study of *FLO1* de-repression in *tup1* and *cyc8* single deletion mutants can therefore lead to unique insights into the de-repression mechanism while being a representative model for other Cyc8–Tup1 repressed genes.

## 4 Discussion

Despite considerable research, the precise mechanism of repression by the Cyc8–Tup1 complex has proved elusive. The complex can form a perplexing range of contacts with numerous binding partners mediated through the WD40 domain of Tup1p and the TPR domain of Cyc8p [64]. Cyc8–Tup1interacts with non-acetylated histone H3 and H4 tails, histone deacetylases and several different RNA polymerase II holoenzyme components, as well as with a large variety of DNA binding factors. This has led to the proposals of several, not mutually exclusive, mechanisms of action. One model proposes that Cyc8–Tup1 alters chromatin structure into a repressive form; another that it inhibits the RNA polymerase II holoenzyme by contacting it; and a third that it blocks transcription activators [7], [8], [23].

Our analysis of public Tup1p occupancy and activity data suggests that different subsets of Cyc8–Tup1 repressed genes may exist, subject to different extents of repression by the various overlapping mechanisms proposed for Cyc8–Tup1 activity. We propose that the *FLO1* gene is representative of a distinct subset of subtelomeric genes strongly repressed by the Cyc8–Tup1 complex. Furthermore, our analysis may be an underestimate of this novel subset of genes since a recent study identified 36 new genes subject to Cyc8–Tup1 repression, 14 of which were located in subtelomeres [53].

We previously mapped a 30-nucleosome array and demonstrated a range of changes in the extended gene-free upstream region of the *FLO1* gene in addition to extensive remodelling at the proximal promoter [41]. These changes, which were attributed to the absence or presence of the Swi–Snf or Cyc8–Tup1 complexes, go beyond current promoter-centred models and raise questions as to how such long-range chromatin remodelling effects are achieved.

Two modes of association have been proposed for the Cyc8–Tup1 complex: a continuous polymerisation along the chromatin fibre [65], or localisation at distinct foci [55]. We confirmed the occupancy of Tup1p and Cyc8p across the *FLO1* promoter and upstream region concentrated at a single location around − 700 bp upstream of the transcription start site within a region of 3.5 kb of upstream sequence analysed [24], [55]. Recruitment of Tup1p to this site required the Cyc8p component of the complex. However, Cyc8p remained associated with the *FLO1* promoter in the absence of Tup1p where it either directly or indirectly imparts partial repression. Interestingly, the peak of Cyc8p detected in the absence of Tup1p was greater than in wt. This could be due to either increased recruitment of Cyc8p in the absence of Tup1p or, most likely, increased epitope availability. Importantly, the data suggest that only the *cyc8* strain is representative of a true Cyc8–Tup1 null mutant at *FLO1*.

Analysis of the *FLO1* promoter DNA sequence on which Cyc8–Tup1 was enriched revealed the presence of potential binding sites of several proteins known to interact or recruit either Tup1p or Cyc8p including Mcm1p, Phd1p, Skn7p and Yap6p [66], [67]. Individual deletion of these genes, or mutation of the Mcm1p DNA binding site, failed to de-repress *FLO1* transcription (data not shown). This suggests that recruitment of Cyc8–Tup1 to *FLO1* either is redundant, involves a scaffold of multiple proteins or occurs via other proteins.

Our indirect end-labelling analysis revealed that Cyc8–Tup1 colocalises with a chromatin hypersensitive site situated within an array of six strongly positioned promoter proximal nucleosomes. ChIP analysis indicated a higher than background nucleosome density at the repressed *FLO1* promoter and upstream region. However, in *cyc8* mutants, the promoter nucleosome positioning is lost resulting in a dramatic expansion of the hypersensitive site and increased restriction enzyme accessibility to the underlying DNA. This is accompanied by a marked decrease in histone occupancy at the proximal promoter immediately adjacent to where Cyc8–Tup1 is normally bound. Furthermore, significant loss in histone density was seen to extend over the entire 3.5 kb upstream region analysed. A general depletion of histones was not the cause of the extensive histone loss as Western blot analysis showed that global histone levels were unaffected in the *cyc8* mutant. However, it is possible that upregulation of unannotated non-coding transcripts mapped upstream of *FLO1* in the *cyc8* mutant could contribute to the upstream histone loss shown [68].

Overall, the changes are consistent with a randomisation or mobilisation of the array of positioned proximal promoter nucleosomes and altered positions and loss of more distal nucleosomes [41]. These data suggest that when bound at the *FLO1* promoter, the Cyc8–Tup1 complex is required to establish or maintain a highly organised repressive chromatin structure encompassing the promoter and upstream region.

Cyc8–Tup1 repression has been linked with histone deacetylation enzymatic activity as well as deacetylated histone binding *in vitro* [7]. Our data show that Cyc8–Tup1 does not bind to any particular region of deacetylated histones at the repressed *FLO1* promoter. However, in the absence of Cyc8–Tup1 we observed increased acetylation of histone H3 and H4 residues across chromatin covering the *FLO1* promoter and upstream region. This suggests that extensive histone acetylation over the *FLO1* promoter and upstream region is counteracted by Cyc8–Tup1.

From a panel of candidate histone deacetylases (HDACs), we identified Hda1p and Rpd3p as contributing redundantly to *FLO1* promoter and upstream region deacetylation and gene repression. Single deletion of Hda1p also increased promoter acetylation but *FLO1* was not de-repressed in this strain. However, deletion of both Rpd3p and Hda1p led to greater promoter histone acetylation compared to levels in either single mutant, and importantly, yielded *FLO1* gene de-repression. The histone hyperacetylation evident in the *rpd3 hda1* double mutant was lower than the hyperacetylation in the absence of Cyc8–Tup1, which correlates with the lower level of de-repression in the *rpd3 hda1* mutant. This suggests that histone deacetylation by Hda1p and Rpd3p at the repressed *FLO1* promoter could be redundant with the activity of other HDACs if a threshold level of histone acetylation is required to enable maximum *FLO1* transcription. Alternatively, the increased histone acetylation in the single *hda1* and double *hda1 rpd3* mutants may lead to poised chromatin, requiring additional promoter de-repression or gene activators to potentiate full transcription.

In the mutant deleted for both Rpd3p and Hda1p, where *FLO1* transcription is partially de-repressed, we observed significant histone depletion across the *FLO1* promoter and upstream region. However, histone loss was greatest in the *cyc8* mutant in which *FLO1* transcription is fully de-repressed. These data suggest that Cyc8–Tup1 and both the HDACs, Rpd3p and Hda1p, contribute to the ordered array of nucleosomes at the repressed *FLO1* gene promoter and upstream region. Interestingly, significant histone loss was detected in the absence of *FLO1* gene de-repression in the *hda1* single mutant. This result highlights that repression of *FLO1* transcription can occur even when the promoter is significantly depleted of histone H3, as well as acetylated. Moreover, these data indicate that nucleosome loss is not a consequence of transcription, but may precede it. Together, the data suggest that a threshold level of nucleosome loss and/or histone acetylation may be required across the *FLO1* promoter and upstream region before maximal *FLO1* transcription can occur.

One model for Cyc8–Tup1 activity purports that it recruits HDACs to target promoters which deacetylate adjacent histones to bring about gene repression [29], [63]. The promoter histone deacetylation has been proposed to enhance further binding of Cyc8–Tup1 thereby reinforcing transcription repression of target genes [31]. In accordance with separate involvements at the *ENA1* and *STE6* promoters [29], [30], we find that the histone deacetylases Hda1p and Rpd3p were centred on the Cyc8–Tup1 binding site at the *FLO1* promoter. Importantly, Hda1p and Rpd3p occupancies at *FLO1* were reduced in a *cyc8* mutant, suggesting that their association with the promoter was Cyc8–Tup1 dependent. Surprisingly, Cyc8–Tup1 occupancy was not reduced in the *rpd3 hda1* double mutant. Although seemingly at odds with the proposed histone deacetylase-mediated enhancement of Cyc8–Tup1 binding, the result may also reflect persistence of the complex at the promoter during gene activation in the *rpd3 hda1* mutant, within the sensitivity limits of the Tup1p ChIP analysis where *FLO1* transcription is only partially de-repressed.

Overall, our data support a role for Cyc8–Tup1 in organising an ordered array of deacetylated nucleosomes at the wild-type *FLO1* promoter and upstream region which is required for gene repression. Our data also indicate a Cyc8–Tup1 dependent role for Hda1p and Rpd3p in establishing or maintaining this repressive chromatin structure.

In our previous study of chromatin remodelling at *FLO1*, the changes in *cyc8* chromatin relative to wild-type were attributed to the actions of the Swi–Snf complex [41]. We therefore examined Swi–Snf occupancy at the *FLO1* promoter and revealed increased levels of the Snf2p subunit of Swi–Snf at the de-repressed *FLO1* promoter in a *cyc8* mutant compared to the repressed wild-type. Additionally, we detected intermediate levels of the Snf2p co-activator at the *FLO1* promoter in the *rpd3 hda1* double mutant where *FLO1* transcription is partially de-repressed, suggesting a correlation between transcription and co-activator binding at *FLO1*. Furthermore, we demonstrate that Snf2p binds maximally to the same region vacated by Cyc8–Tup1 in the *cyc8* mutant, and may show partial co-occupancy with Cyc8–Tup1 in the *rpd3 hda1* mutant.

Based on our data and that of others, one model for regulation of *FLO1* transcription might involve a dynamic equilibrium between the abundant HDACs, HATs, Swi–Snf and Cyc8–Tup1 complexes which can be biased one way or another to either promote gene repression or activation. In wt cells, Cyc8–Tup1 dependent enrichment of Rpd3p and Hda1p at *FLO1* would promote strongly positioned deacetylated nucleosomes across the promoter and upstream region and repress gene transcription (Fig. 8C, wt). In the absence of Cyc8–Tup1, reduced Rpd3p and Hda1p occupancy would allow HAT activity to yield histone hyper-acetylation. The high histone acetylation would enable Swi–Snf to bind the *FLO1* promoter and initiate extensive histone eviction leading to transcription (Fig. 8C, *cyc8*) [69], [70], [71], [72].

In the *rpd3 hda1* mutant, the equilibrium between HDAC and HAT binding would again be upset, allowing increased HAT occupancy at *FLO1*. The subsequent increased histone acetylation would enable Swi–Snf to drive histone eviction and de-repress *FLO1* transcription. However, other HDACs could partially compensate for the loss of Rpd3p and Hda1p. In conjunction with Cyc8–Tup1 which remains bound at *FLO1*, full HAT binding at *FLO1* would therefore be restricted resulting in partial histone acetylation, reduced Swi–Snf binding, lower histone eviction and reduced transcription de-repression (Fig. 8C, *rpd3 hda1*).

## 5 Concluding remarks

In this study, we confirmed that Cyc8–Tup1 binds to a distinct site at the *FLO1* promoter where it is associated with an array of strongly positioned, deacetylated nucleosomes covering the promoter and upstream region. We revealed that in the absence of Cyc8–Tup1, there is a gross remodelling of *FLO1* promoter and upstream chromatin involving nucleosome acetylation, rearrangement and eviction which accompanies *FLO1* gene de-repression. We demonstrate direct involvement of Swi–Snf in this remodelling whereby Swi–Snf is recruited in the *cyc8* mutant to the same site previously occupied by Cyc8–Tup1. Hence, the extensively remodelled *FLO1* promoter and upstream chromatin region is under the control of chromatin remodellers bound at a single discrete site. These findings rule out a propagating mechanism for the activities of Swi–Snf and Cyc8–Tup1, and suggest that the long-range impact on modulation of *FLO1* promoter and upstream chromatin may be due to the large size of the participating complexes, or involves the three-dimensional packing of the nucleosomal array.

Intriguingly, the histone acetylation which accompanies *FLO1* transcription emanates extensively from the Tup1p peak where it marks the region in which the extensive changes in chromatin structure are observed. It is therefore possible that part of the antagonistic activity of the Cyc8–Tup1 and Swi–Snf complexes is acted out at the level of histone acetylation, and that the rapid reversibility of this modification allows for the dynamic switch between gene activation and repression.

Our analysis of genomic data suggest that genes strongly de-repressed in the absence of *TUP1* share a significantly increased involvement of Hda1p in the UAS, and also have in common a high incidence of nucleosome depletion upon de-repression in an extensive upstream region. The Cyc8–Tup1 mechanism for repression and de-repression observed at *FLO1* is therefore representative of this larger subset. Further investigation of the various classes of Tup1-Cyc8 bound and regulated promoters might reveal alternative repression mechanisms and help to reconcile the different mechanisms that have been proposed.

The following are the Supplementary data related to this article.

Table S1 *S. cerevisiae* strains.

Table S2 Primers used for qPCR.

Fig. S1 Western blot analysis of Tup1p and Myc-Cyc8p levels in wt (*CYC8-myc*), *cyc8* and *tup1* (*CYC8-myc*) strains. The results confirm that Myc-Cyc8p levels are similar to native Cyc8p levels in the wt (*CYC8-myc*) and *tup1* (*CYC8-myc*) strains. In addition, Tup1p abundance is unaltered in the *CYC8-myc* strain.

Fig. S2 Accessibility in chromatin of underlying DNA sequence is enhanced in hypersensitive regions. Restriction site accessibility in wt and *cyc8* nuclei for the enzymes and concentrations indicated. (**A**) Southern blot of agarose gel separation of restriction fragments hybridised consecutively for the 0 to − 1 kb upstream region (top, probed relative to *Spe*I site at –1284 bp) and − 2 kb to − 3 kb region (bottom, probed relative to *Spe*I site at –1529 bp). Hypersensitive regions in *cyc8* chromatin (top) were more susceptible to restriction enzymes (as well as endonucleases). (**C**) Further collection of digests probed for the − 2 kb to − 3 kb region relative to *Rsa*I site at –1660 bp. (**B**) Graph plotting fragment intensity ratios to give fold increase accessibility in *cyc8* compared to wt nuclei at various sites (♦200 U/ml, □1000 U/ml). Absolute values of accessibility within the core − 700 bp hypersensitive site were constitutively high in both strains, giving a lower ratio than in adjacent regions. Accessibility at most sites was substantially increased in *cyc8* strains (ranging from less than 15% to more than 75% cleavage, not shown) except around the − 700 bp hypersensitive site, where it was already much higher in wild-type strains. Yet limit digests (when no further cutting could be achieved using higher concentrations of restriction endonuclease) did not reach the 80%–95% cleavage that might be expected of naked DNA [48].

Fig. S3 **(A)** ChIP analysis of Myc-Hda1p and Myc-Rpd3p occupancy at the *ENA1* and *INO1* promoters. Myc-Hda1p and Myc-Rpd3p occupancy measured in wt by ChIP analysis at the *ENA1* and *INO1* promoters which were used as positive binding controls. For the Myc-Hda1p and Myc-Rpd3p ChIP analysis, cells were sequentially cross-linked with ethylene glycol bis[succinimidyl succinate] (EGS) and formaldehyde as described in Materials and Methods. Occupancies at each site were expressed as the ratio of IP/input and normalised to the IP/input ratio from a strain containing untagged HDAC proteins. **(B)** Comparison of Tup1p ChIP analysis using either formaldehyde or EGS and formaldehyde as the cross-linking reagents. Tup1p ChIP analysis was performed in wt cells cross-linked with either formaldehyde (CH_2_O) or after sequential EGS and formaldehyde cross-linking. Tup1p occupancy at *RNR2* and *STE6* in the differently treated cells was compared. The results show that the use of the EGS did not alter the binding profile of Tup1p at *RNR2* and *STE6* promoters which were used as positive and negative Tup1p binding sites respectively.

Fig. S4 Snf2 proteins are concentrated at a single location at the de-repressed *FLO1* gene promoter. **(A)** Diagram of the amplicons used in chromatin immunoprecipitation analysis covering a region up to 1800 base pairs upstream (− 1800), labelled by the distance (in bp) from their midpoints to the *FLO1* translation start site (+ 1). Cross-linked chromatin fragments from wild-type (wt), *CYC8* and *RPD3 HDA1* deleted strains (*cyc8* and *rpd3 hda1* respectively) were immunoprecipitated with antibodies against Snf2p and the DNA content analysed by qPCR. Occupancies were normalised to the *TEL-VI* control region. The results represent the average from three to four independent experiments with bars representing SEM. The data from the amplicon centred around the − 585 bp *FLO1* promoter region are shown in Fig. 7C. **(B)** Myc-Snf5p occupancy at the − 585 bp *FLO1* promoter region was also measured by ChIP analysis in wt and *cyc8* strains harbouring genomic copies of *SNF5* tagged with a 9-myc epitope alongside relevant controls. Occupancies were normalised to the *TEL-VI* region as a control. The results represent the average from three independent experiments, with bars representing SEM and support the Snf2p ChIP data.

## Author Contributions

Conceived and designed the experiments: ABF YT SP. Performed the experiments: ABF SB MC. Contributed reagents/materials/analysis: ABF SB MC YT SP. Wrote the paper: ABF SP.

## Funding

This work was funded by the 10.13039/100004440Wellcome Trust [grant number 092533/Z/10/Z to ABF and grant number 045117 to SP]. SP acknowledges 10.13039/501100000268BBSRC funding. Funding for open access charge: [10.13039/100004440Wellcome Trust/092533/Z/10/Z].
