# Supplementary material for: The yeast Cyc8–Tup1 complex cooperates with Hda1p and Rpd3p histone deacetylases to robustly repress transcription of the subtelomeric FLO1 gene
Source: Biochim Biophys Acta Gene Regul Mech. 2014 Nov;1839(11):1242–55. doi: 10.1016/j.bbagrm.2014.07.022 (PMC4316177; doi:10.1016/j.bbagrm.2014.07.022)
Supplement: Table S1 — S. cerevisiae strains. [file mmc1.doc]

**Table S1. *S. cerevisiae* strains**

| Strain | Genotype | Source |
| --- | --- | --- |
| BY4741 | *Mat*a*; his3Δ1; leu2Δ0; met15Δ0; ura3Δ0* | Open Biosystems |
| *rpd3* *hda1* | *Mat*a*; his3Δ1; leu2Δ0; met15Δ0; ura3Δ0, rpd3 Δ::URA3*; *hda1 Δ::KanMX4* | This study |
| *tup1* | *Mat*a*; his3Δ1; leu2Δ0; met15Δ0; ura3Δ0;* *tup1Δ::KanMX4* | Open Biosystems |
| *cyc8* | *Mat*a; *his3Δ1; leu2Δ0; met15Δ0; ura3Δ0*; *cyc8Δ::KanMX4* | Open Biosystems |
| *rpd3* | *Mat*a; *his3Δ1; leu2Δ0; met15Δ0; ura3Δ0*; *rpd3Δ::KanMX4* | Open Biosystems |
| *hda1* | *Mat*a; *his3Δ1; leu2Δ0; met15Δ0; ura3Δ0*; *hda1Δ::KanMX4* | Open Biosystems |
| *hos1* | *Mat*a; *his3Δ1; leu2Δ0; met15Δ0; ura3Δ0*; *hos1Δ::KanMX4* | Open Biosystems |
| *hos2* | *Mat*a; *his3Δ1; leu2Δ0; met15Δ0; ura3Δ0*; *hos2Δ::KanMX4* | Open Biosystems |
| *sir2* | *Mat*a; *his3Δ1; leu2Δ0; met15Δ0; ura3Δ0*; *sir2Δ::KanMX4* | Open Biosystems |
| *hst1* | *Mat*a; *his3Δ1; leu2Δ0; met15Δ0; ura3Δ0*; *hst1Δ::KanMX4* | Open Biosystems |
| *rpd3 hos2* | *Mat*a; *his3Δ1; leu2Δ0; met15Δ0; ura3Δ0*; *hos2Δ::KanMX4; rpd3::URA3* | This study |
| *rpd3 hos1* | *Mat*a; *his3Δ1; leu2Δ0; met15Δ0; ura3Δ0*; *hos1Δ::KanMX4; rpd3 Δ::URA3* | This study |
| *hos1 hos2* | *Mat*a; *his3Δ1; leu2Δ0; met15Δ0; ura3Δ0*; *hos1Δ::KanMX4; hos2Δ::URA3* | This study |
| *rpd3 hos1 hos2* | *Mat*a; *his3Δ1; leu2Δ0; met15Δ0; ura3Δ0*; *hos1Δ::KanMX4; rpd3 Δ::URA3; hos2 Δ::HIS3* | This study |
| YSB01 #9 | *MAT*a; *his3Δ1; leu2Δ0; met15Δ0; ura3Δ0*; *RPD3*-*9Myc::KanMX4* | This study |
| YSB02 #2 | *MAT*a; *his3Δ1; leu2Δ0; met15Δ0; ura3Δ0*; *HDA1*-*9Myc::KanMX4* | This study |
| YSB03 #8 | *MAT*a; *his3Δ1; leu2Δ0; met15Δ0; ura3Δ0;* *RPD3*-9Myc*::KanMX4;* *cyc8Δ::URA3* | This study |
| YSB04 #1 | *MAT*a; *his3Δ1; leu2Δ0; met15Δ0; ura3Δ0;* *HDA1*-9Myc*::KanMX4;* *cyc8Δ::URA3* | This study |
| YPOD1 | *MAT*a*; his3Δ1; leu2Δ0; met15Δ0; ura3Δ0; CYC8-9Myc::KanMX4* | This study |
| YMC11 | *MAT*a*; his3Δ1; leu2Δ0; met15Δ0; ura3Δ0; CYC8-9Myc::KanMX4; tup1::URA3* | This study |
