# Supplementary material for: The yeast Cyc8–Tup1 complex cooperates with Hda1p and Rpd3p histone deacetylases to robustly repress transcription of the subtelomeric FLO1 gene
Source: Biochim Biophys Acta Gene Regul Mech. 2014 Nov;1839(11):1242–55. doi: 10.1016/j.bbagrm.2014.07.022 (PMC4316177; doi:10.1016/j.bbagrm.2014.07.022)
Supplement: Table S2 — Primers used for qPCR. [file mmc2.doc]

**Table S2. Primers used for qPCR**

| **Primer** | **Sequence** | **Description (amplicon)** |
| --- | --- | --- |
| IPFLO1-F | AAAGGAACATATTTCACTCTTGCTC | ChIP (*FLO1*, -118) |
| IPFLO1-R | TCTGTTTACTGGTGACAAGAATTAAAA | ChIP (*FLO1*, -118) |
| IPFLO2-F | TGTGGAACCTTCTACAGTACTTCGG | ChIP (*FLO1*, -360) |
| IPFLO2-R | TTTGAGTGCCTTTCAACAATTTCAGACTT | ChIP (*FLO1*, -360) |
| IPFLO3-F | GCTTCCAGTATGCTTTCACG | ChIP (*FLO1*, -585) |
| IPFLO3-R | GCCTACGTATTCTCCGTCAC | ChIP (*FLO1*, -585) |
| IPFLO4-F | AGTCTCATTACCTAAACGCCAG | ChIP (*FLO1*, -904) |
| IPFLO4-R | CTGAAACTGGCTAGCATAACAC | ChIP (*FLO1*, -904) |
| IPFLO5-F | TTGAATGGCACTAGTCGATCG | ChIP (*FLO1*, -1240) |
| IPFLO5-R | TTAAACTTACGGCATCTTGAACATT | ChIP (*FLO1*, -1240) |
| IPFLO6-F | GGGAAACACAGAAAACACTCC | ChIP (*FLO1*, -1628) |
| IPFLO6-R | GGTGAGCAACGTCTAGGTC | ChIP (*FLO1*, -1628) |
| IPFLO7-F | TTGCCTTCATGACCCACC | ChIP (*FLO1*, -2297) |
| IPFL07-R | AGAACTCAACTATGCTGCTGG | ChIP (*FLO1*, -2297) |
| IPFLO8-F | AGATTCCATTGTTGAATGTGACACG | ChIP (*FLO1*, -3333) |
| IPFLO8-R | CTAAACCAGGTATGGCCTAGAGT | ChIP(*FLO1*, -3333) |
| IPRNR2-F | CGACAACTATGCGAAATCCG | ChIP (*RNR2*) |
| IPRNR2-R | ACATTGTACCCATGCCCG | ChIP (*RNR2*) |
| AF-IPSTE6-F | GATATGGCTGAACTATCTCCCG | ChIP (*STE6*) |
| AF-IPSTE6-R | GCTTGTTCTTTGTTTCCTAGTGG | ChIP (*STE6*) |
| FLO1RT-F | TACCACCACAGACGGGTTCT | RT-qPCR (*FLO1* ORF) |
| FLO1RT-R | CAACAGTTGAACGCGGTTGC | RT-qPCR (*FLO1* ORF) |
| ACT1ORF318F | GAGGTTGCTGCTTTGGTTATTGA | ChIP and RT-qPCR (*ACT1*) |
| ACT1ORF318R | ACCGGCTTTACACATACCAGAAC | ChIP and RT-qPCR (*ACT1*) |
| TEL VI-R121F | CGTGTGTAGTGATCCGAACTCAGT | ChIP normalization (TEL) |
| TEL VI-R121R | GACCCAGTCCTCATTTCCATCAATAG | ChIP normalization (TEL) |
| Int-V-F | TAAGAGGTGATGGTGATAGGCGT | ChIP normalization (IntV) |
| Int-V-R | CCCTCGGGTCAAACACTACAC | ChIP normalization (IntV) |
| INO1Prom-F | GATGCGGAATCGAAAGTGTT | ChIP (*INO1*) |
| INO1Prom-R | TCTGGGAAAGAAGGATGAAGA | ChIP (*INO1*) |
| ENA1-Prom-F | CTGCACGAAGTGGTTACATTG | ChIP (*ENA1*) |
| ENA1-Prom-R | CAAGGAAAGAGGCAATAATTCG | ChIP (*ENA1*) |
