# Supplementary figures and images for: The yeast Cyc8–Tup1 complex cooperates with Hda1p and Rpd3p histone deacetylases to robustly repress transcription of the subtelomeric FLO1 gene
Source: Biochim Biophys Acta Gene Regul Mech. 2014 Nov;1839(11):1242–55. doi: 10.1016/j.bbagrm.2014.07.022 (PMC4316177; doi:10.1016/j.bbagrm.2014.07.022)

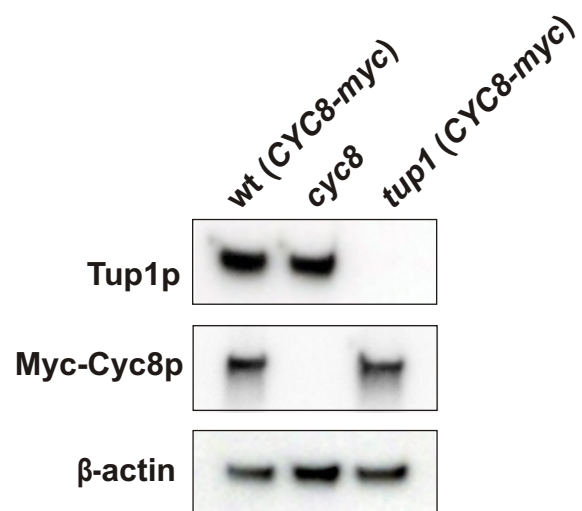

**Supplemental Figure S1**

Supplement: Fig. S1 — Western blot analysis of Tup1p and Myc-Cyc8p levels in wt (CYC8-myc), cyc8 and tup1 (CYC8-myc) strains. The results confirm that Myc-Cyc8p levels are similar to native Cyc8p levels in the wt (CYC8-myc) and tup1 (CYC8-myc) strains. In addition, Tup1p abundance is unaltered in the CYC8-myc strain. [file mmc3.pdf]

**A**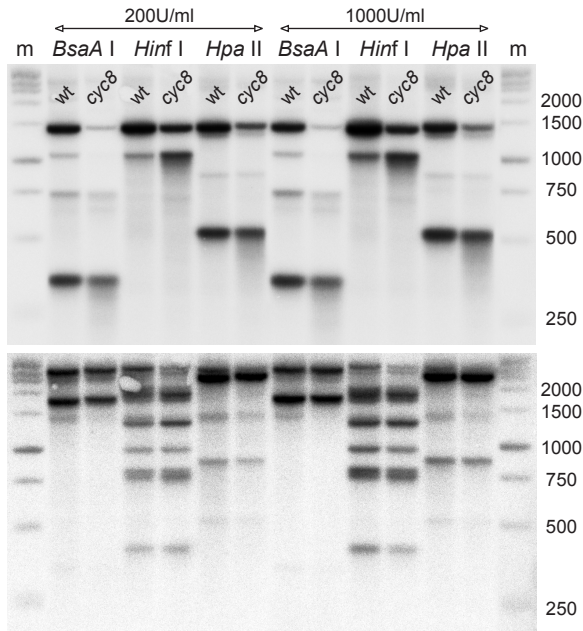**B**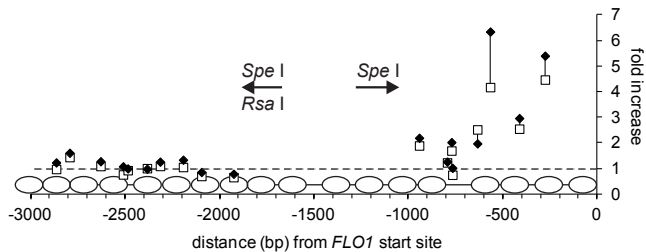**C**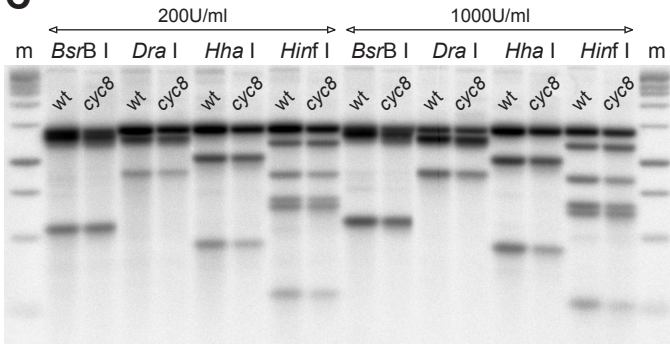**Supplemental Figure S2**

Supplement: Fig. S2 — Accessibility in chromatin of underlying DNA sequence is enhanced in hypersensitive regions. Restriction site accessibility in wt and cyc8 nuclei for the enzymes and concentrations indicated. (A) Southern blot of agarose gel separation of restriction fragments hybridised consecutively for the 0 to − 1 kb upstream region (top, probed relative to SpeI site at –1284 bp) and − 2 kb to − 3 kb region (bottom, probed relative to SpeI site at –1529 bp). Hypersensitive regions in cyc8 chromatin (top) were more susceptible to restriction enzymes (as well as endonucleases). (C) Further collection of digests probed for the − 2 kb to − 3 kb region relative to RsaI site at –1660 bp. (B) Graph plotting fragment intensity ratios to give fold increase accessibility in cyc8 compared to wt nuclei at various sites (♦200 U/ml, □1000 U/ml). Absolute values of accessibility within the core − 700 bp hypersensitive site were constitutively high in both strains, giving a lower ratio than in adjacent regions. Accessibility at most sites was substantially increased in cyc8 strains (ranging from less than 15% to more than 75% cleavage, not shown) except around the − 700 bp hypersensitive site, where it was already much higher in wild-type strains. Yet limit digests (when no further cutting could be achieved using higher concentrations of restriction endonuclease) did not reach the 80%–95% cleavage that might be expected of naked DNA [48]. [file mmc4.pdf]

**A****Myc-HDAC ChIP**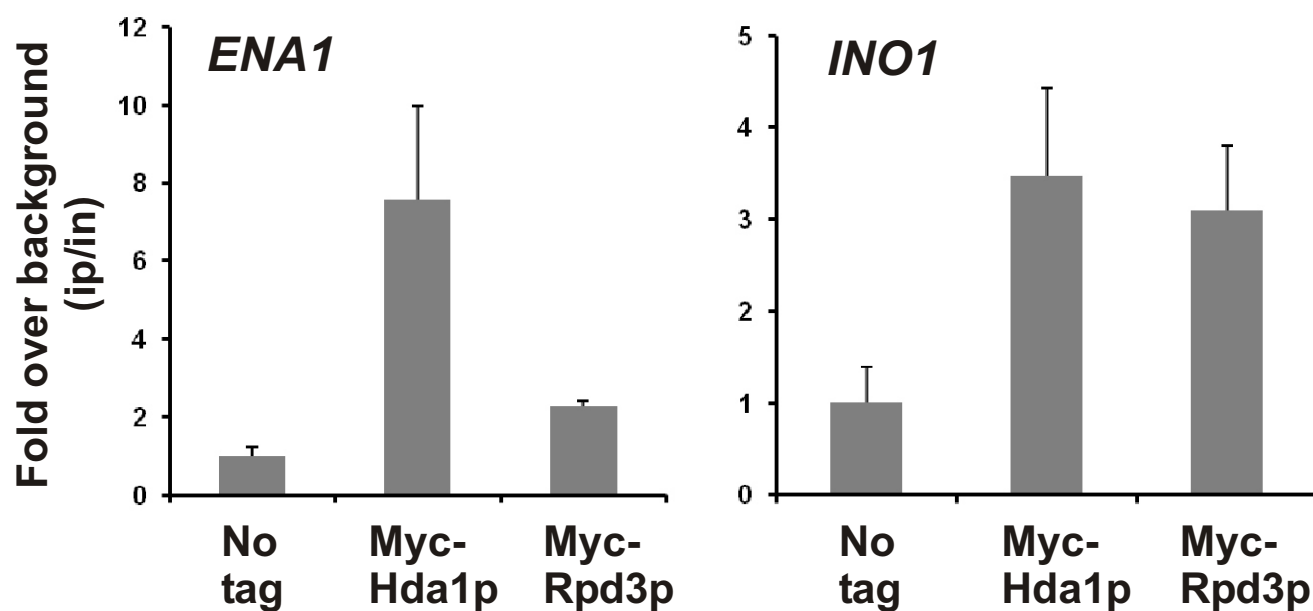**B****Tup1p ChIP**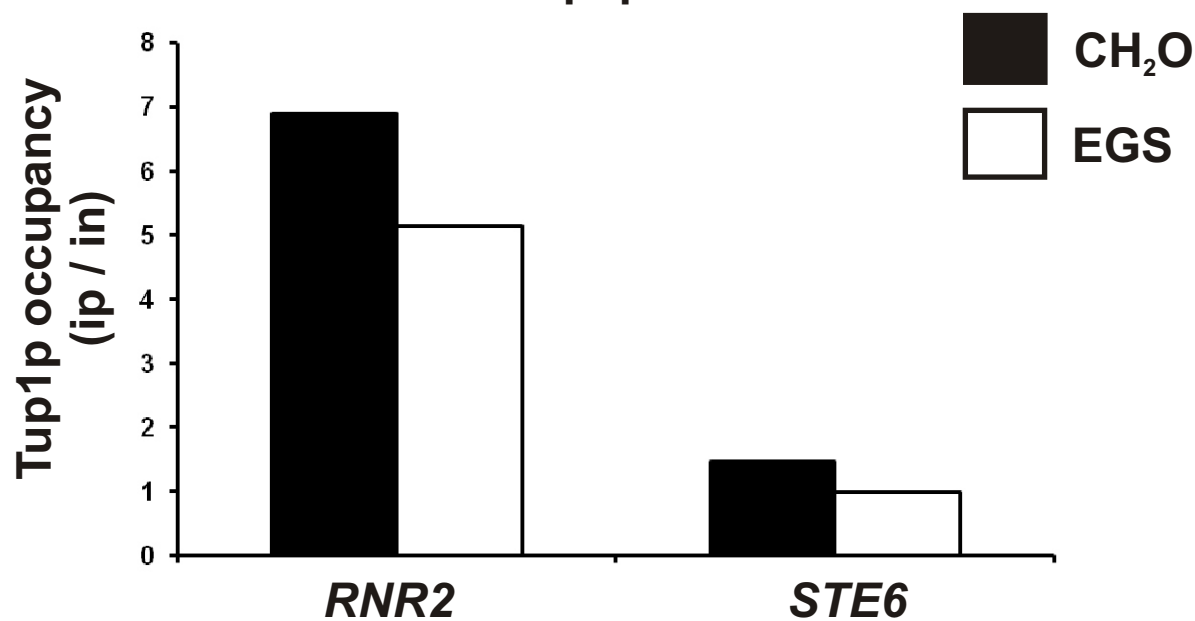

Supplement: Fig. S3 — (A) ChIP analysis of Myc-Hda1p and Myc-Rpd3p occupancy at the ENA1 and INO1 promoters. Myc-Hda1p and Myc-Rpd3p occupancy measured in wt by ChIP analysis at the ENA1 and INO1 promoters which were used as positive binding controls. For the Myc-Hda1p and Myc-Rpd3p ChIP analysis, cells were sequentially cross-linked with ethylene glycol bis[succinimidyl succinate] (EGS) and formaldehyde as described in Materials and Methods. Occupancies at each site were expressed as the ratio of IP/input and normalised to the IP/input ratio from a strain containing untagged HDAC proteins. (B) Comparison of Tup1p ChIP analysis using either formaldehyde or EGS and formaldehyde as the cross-linking reagents. Tup1p ChIP analysis was performed in wt cells cross-linked with either formaldehyde (CH2O) or after sequential EGS and formaldehyde cross-linking. Tup1p occupancy at RNR2 and STE6 in the differently treated cells was compared. The results show that the use of the EGS did not alter the binding profile of Tup1p at RNR2 and STE6 promoters which were used as positive and negative Tup1p binding sites respectively. [file mmc5.pdf]

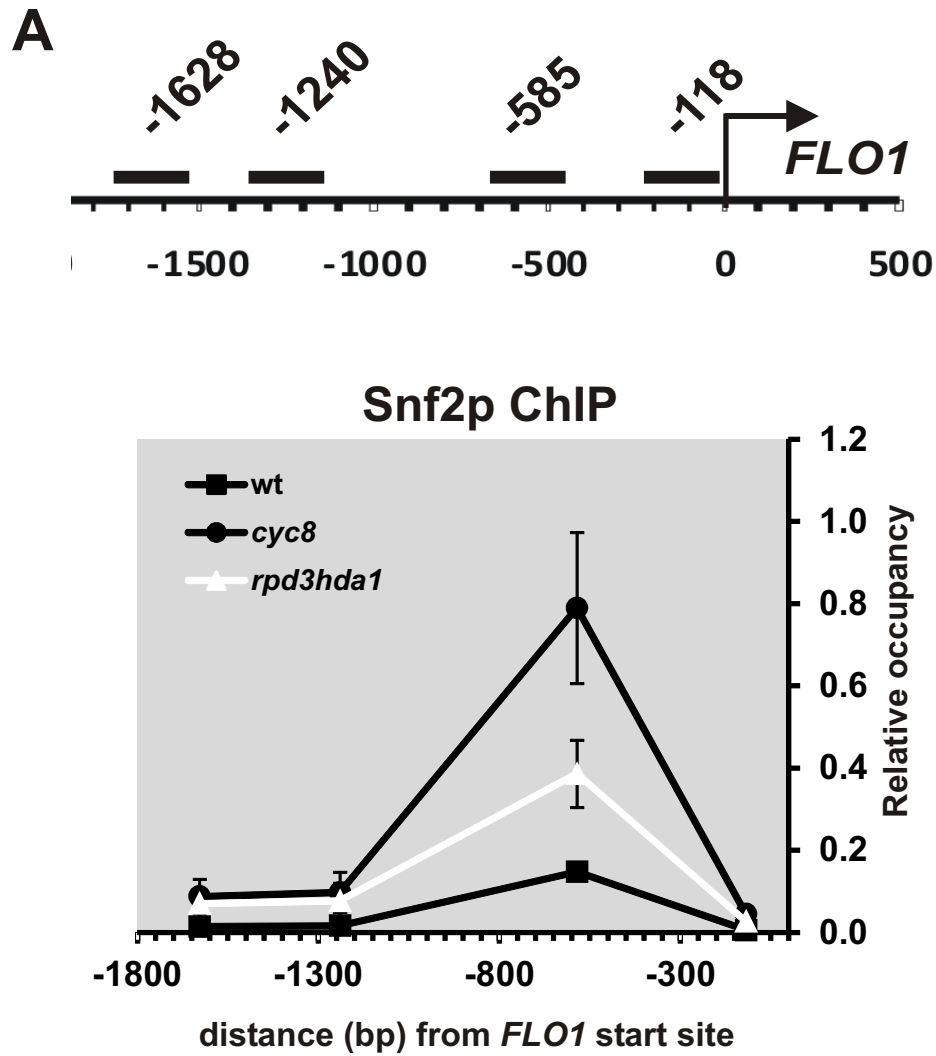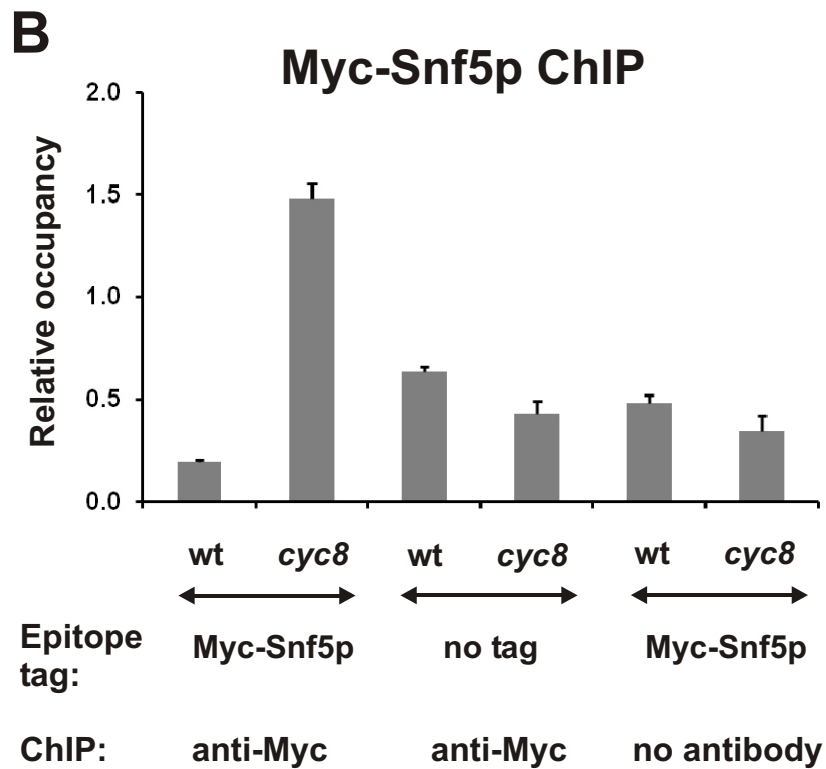

Supplemental Figure S4

Supplement: Fig. S4 — Snf2 proteins are concentrated at a single location at the de-repressed FLO1 gene promoter. (A) Diagram of the amplicons used in chromatin immunoprecipitation analysis covering a region up to 1800 base pairs upstream (− 1800), labelled by the distance (in bp) from their midpoints to the FLO1 translation start site (+ 1). Cross-linked chromatin fragments from wild-type (wt), CYC8 and RPD3 HDA1 deleted strains (cyc8 and rpd3 hda1 respectively) were immunoprecipitated with antibodies against Snf2p and the DNA content analysed by qPCR. Occupancies were normalised to the TEL-VI control region. The results represent the average from three to four independent experiments with bars representing SEM. The data from the amplicon centred around the − 585 bp FLO1 promoter region are shown in Fig. 7C. (B) Myc-Snf5p occupancy at the − 585 bp FLO1 promoter region was also measured by ChIP analysis in wt and cyc8 strains harbouring genomic copies of SNF5 tagged with a 9-myc epitope alongside relevant controls. Occupancies were normalised to the TEL-VI region as a control. The results represent the average from three independent experiments, with bars representing SEM and support the Snf2p ChIP data. [file mmc6.pdf]
